# Antigenic characterization of highly pathogenic avian influenza A(H5N1) viruses with chicken and ferret antisera reveals clade-dependent variation in hemagglutination inhibition profiles

**DOI:** 10.1038/s41426-018-0100-7

**Published:** 2018-05-31

**Authors:** Diep Thi Nguyen, Samuel S. Shepard, David Francis Burke, Joyce Jones, Sharmi Thor, Long Van Nguyen, Tho Dang Nguyen, Amanda Balish, Dang Nguyen Hoang, Thanh Long To, Munir Iqbal, David E. Wentworth, Erica Spackman, H Rogier van Doorn, C. Todd Davis, Juliet E. Bryant

**Affiliations:** 1 National Center for Veterinary Diagnostics, Department of Animal Health, Hanoi, Vietnam; 2 Department of Animal Health, Ministry of Agriculture and Rural Development of Vietnam, Hanoi, Vietnam; 3 Oxford University Clinical Research Unit, Hanoi, Vietnam; 4 Center for Tropical Medicine and Global Health, Nuffield Department of Clinical Medicine, University of Oxford, Oxford, UK; 5 Influenza Division, Centers for Disease Control and Prevention, Atlanta, GA 30333 USA; 6 Department of Zoology, Cambridge University, Cambridge, UK; 7 The Pirbright Institute, Pirbright, UK; 8 United States Department of Agriculture, Southeast Poultry Research Laboratory, Athens, GA USA

## Abstract

Highly pathogenic avian influenza (HPAI) A(H5N1) viruses pose a significant economic burden to the poultry industry worldwide and have pandemic potential. Poultry vaccination against HPAI A(H5N1) viruses has been an important component of HPAI control measures and has been performed in Vietnam since 2005. To systematically assess antigenic matching of current vaccines to circulating field variants, we produced a panel of chicken and ferret antisera raised against historical and contemporary Vietnamese reference viruses representing clade variants that were detected between 2001 and 2014. The antisera were used for hemagglutination inhibition (HI) assays to generate data sets for analysis by antigenic cartography, allowing for a direct comparison of results from chicken or ferret antisera. HI antigenic maps, developed with antisera from both hosts, revealed varying patterns of antigenic relationships and clustering of viruses that were dependent on the clade of viruses analyzed. Antigenic relationships between existing poultry vaccines and circulating field viruses were also aligned with in vivo protection profiles determined by previously reported vaccine challenge studies. Our results establish the feasibility and utility of HPAI A(H5N1) antigenic characterization using chicken antisera and support further experimental and modeling studies to investigate quantitative relationships between genetic variation, antigenic drift and correlates of poultry vaccine protection in vivo.

## Introduction

Antigenic drift is the Achilles’ heel of designing effective vaccines against rapidly evolving pathogens, such as influenza viruses. In poultry vaccination programs, the challenges are particularly acute when significant genetic variation exists among co-circulating strains, as is the case for highly pathogenic avian influenza (HPAI) A(H5N1) viruses. Antigenic matching, which refers to the antigenic similarity between a given vaccine strain and circulating field viruses, is typically measured by raising antisera in animal models and subsequently comparing antibody-to-antigen reactivity titers of the vaccine strain (homologous titer) vs. circulating field viruses (heterologous titers). For many decades, human seasonal influenza virus antigenic matching has relied on hemagglutination inhibition (HI) assays using ferret antisera as the gold standard for measuring variation among viruses^1,2^. Twice each year, the World Health Organization (WHO) Global Influenza Surveillance and Response System (GISRS), in conjunction with Collaborating Centres for Influenza and Essential Regulatory Laboratories, produces genetic and antigenic analyses of viruses to systematically review diversity of influenza B and influenza A viruses (IAVs) from the Northern and Southern hemispheres, and recommends the antigen formulation for human seasonal influenza vaccines. The recommendations also include a selection of specific candidate vaccine viruses (CVVs) for non-seasonal influenza virus subtypes and genetic lineages as a precautionary measure to produce at-the-ready vaccine seed viruses for pandemic vaccine preparedness^3^.

Ferrets are the preferred animal model to study the susceptibility, virulence and transmission of IAVs because they are highly susceptible to both human and potentially zoonotic IAVs and experience similar clinical outcomes and immune responses to those of humans^4^. However, the production of ferret antisera is expensive and requires animal biocontainment laboratories, which is needed in part to prevent unwanted exposure of naive ferrets to circulating seasonal viruses. The importation of serologically influenza-naive (and specific pathogen free) ferrets into Vietnam is complicated due to customs procedures and the likelihood of infection during transport. Available animal containment facilities within Vietnam are also limited in space and resources, and there are currently no commercial entities that raise laboratory animals under containment for research purposes. Thus, the ferret model of infection is currently unavailable in Vietnam, and representative panels of ferret antisera are rarely available. Because chickens are the principle target for HPAI poultry vaccination and are readily available, they are an ideal animal model for producing reference antisera to evaluate antigenic variation among viruses and vaccines, host immune responses, and vaccine efficacy^5^. In this study, we aimed to produce antisera in chickens against representative HPAI A(H5N1) clade variants at the National Centre for Veterinary Diagnostics (Hanoi, Vietnam) and to use these antisera to characterize circulating HPAI A(H5N1) Vietnamese viruses by HI assay and antigenic cartography. We compared antigenic relationships among viruses using panels of ferret versus chicken antisera to explore the utility and feasibility of HI testing with chicken antisera to antigenically match currently available poultry vaccines used in Vietnam with circulating strains.

## Results

### Collection and preliminary analysis of chicken antisera

The average volume of antiserum collected from individual chickens was 6.5 ml (ranging from 4 to 13 ml). Mean HI titers induced by heterologous viruses varied from 1:40 to 1:640 (Table 1). In ferrets, homologous HI titers varied from 1:160 to 1:1280, while in chickens, homologous HI titers varied from 1:40 to 1:1280 (Table 1). For 4 of 14 viruses (29%), the homologous HI titers raised in chickens and ferrets were equivalent (with titers ranging from 1:320 to 1:1280). For 8 of 14 viruses (57%), the homologous virus HI titer raised in ferrets was higher than that obtained in chickens (ranging from 2- to 8-fold higher), while for 2 of 14 viruses (14%), the antisera raised in chickens yielded higher homologous HI titers (ranging from 2- to 4-fold differences, see Table 1).

**Table 1:** Chicken and ferret antisera produced for this study

	Isolate name	Abbreviation	Clade	Chicken antisera		Ferret sera	
				Number of lots^a^ (volume ml)	HI titer GMT (range)	Homologous HI titer	HI fold difference (ferret HI titer/ mean chicken HI titer)^b^
1	A/goose/Guangdong/1/1996	GD/1/1996	0	2 (7)	80 (80–80)	640	8
2	A/Vietnam/1203/2004	VN/1203/04	1	536	304 (80–640)	320	1
3	A/duck/Vietnam/NCVD-016/2007	DK/VN/016/07	1.1	5 (38)	40 (40–40)		
4	A/Cambodia/R0405050/2007	CB/R0405050/07	1.1	4 (34)	40 (40–40)		
5	A/chicken/Vietnam/NCVD-1192/2012	CK/VN/1192/12	1.1	5 (29)	144 (80–160)		
6	A/Indonesia/5/2005 (CDC-RG2)	INDO/5/05 RG2	2.1.3.2	4 (36)	160 (160–160)	1280	8
7	A/turkey/Turkey/1/2005	TK/1/05	2.2.1	4 (32)	260 (80–320)	640	2
8	A/Egypt/321-Namru3/2007	EG/321/07	2.2.1	5 (35)	160 (160–160)	160	1
9	A/Egypt/321-Namru3/2007 (IDCDC-RG11)	EG/321/07 RG11	2.2.1	3 (26)	40 (40–40)		
10	A/Egypt/N03072/2010 (IDCDC-RG29)	EG/N03072/10 RG29	2.2.1	5 (37)	288 (160–320)	1280	4
11	A/common magpie/Hong Kong/5052/2007	CM/HK/5052/07	2.3.2.1	4 (28)	80 (80–80)		
12	A/Hubei/1/2010 (IDCDC-RG30)	HB/1/10 RG30	2.3.2.1a	5 (22)	80 (80–80)	640	8
13	A/chicken/Vietnam/NCVD-675/2011	CK/VN/675/11	2.3.2.1a	5 (30)	136 (40–160)		
14	A/duck/Vietnam/NCVD-1207/2012	DK/VN/1207/12	2.3.2.1a	5 (34)	80 (80–80)		
15	A/barn-swallow/Hong Kong/D10-1161/2010	BS/HK/1161/10	2.3.2.1b	5 (26)	72 (40–80)	160	2
16	A/duck/Vietnam/NCVD-672/2011	DK/VN/672/11	2.3.2.1b	4 (29)	420 (80–640)	160	4^c^
17	A/duck/Vietnam/NCVD-1163/2012	DK/VN/1163/12	2.3.2.1b	5 (36)	136 (40–160)		
18	A/bar-headed goose/Mongolia/X53/2009	BG/MG/X53/09	2.3.2.1c	3 (17)	745 (320–1280)		
19	A/Hong Kong/6841/2010	HK/6841/10	2.3.2.1c	5 (34)	288 (80–640)	640	1
20	A/duck/Vietnam/NCVD-1648/2012	DK/VN/1648/12	2.3.2.1c	5 (32)	256 (160–320)		
21	A/duck/Vietnam/NCVD-1544/2012	DK/VN/1544/12	2.3.2.1c	5 (33)	288 (80–640)	160	2^c^
22	A/duck/Vietnam/NCVD-003/2008	DK/VN/003/08	2.3.4	5 (41)	80 (80–80)		
23	A/duck/Vietnam/NCVD-391/2009	DK/VN/391/09	2.3.4	5 (27)	80 (80–80)		
24	A/Japanese-white-eye/Hong Kong/1038/06	JW/HK/1038/06	2.3.4	5 (30)	240 (80–320)		
25	A/chicken/India/NIV33487/2006 (IBCDC-RG7)	CK/IND/NIV33487/06 RG7	2.2	5 (26)	80 (80–80)		
26	A/Anhui/1/2005	ANH/1/05	2.3.4	5 (31)	128 (80–160)	640	4
27	A/duck/Vietnam/NCVD-293/2009	DK/VN/293/09	2.3.4.1	4 (24)	80 (80–80)	640	8
28	A/chicken/Vietnam/NCVD-35/2008	CK/VN/35/08	2.3.4.2	5 (32)	160		
29	A/chicken/Vietnam/NCVD-279/2009	CK/VN/279/09	2.3.4.3	4 (25)	70 (40–80)		
30	A/chicken/Vietnam/NCVD-016/2008	CK/VN/016/08	7.1	3 (15)	80 (80–80)		
31	A/chicken/Vietnam/NCVD-3/2008 (IDCDC-RG25A)	CK/VN/3/08 RG25A	7.1	4 (27)	560 (320–640)	640	1
32	A/goose/Guangdong/1/1996 (Re-1)	Re-1 vaccine	0	5 (31)	136 (40–160)		
33	A/duck/Anhui/1/2006 (Re-5)	Re-5 vaccine	2.3.4	5 (26)	144 (80–160)		
34	A/duck/Guangdong/S1322/2010 (Re-6)	Re-6 vaccine	2.3.2.1b	5 (34)	144 (80–160)		

^a^Each lot represents sera collected from an individual chicken

^b^Mean chicken HI titer rounded to nearest serial dilution to calculate HI fold difference

^c^HI fold difference (mean chicken HI titer/ferret HI titer)

### Antigenic analysis of HPAI A(H5N1) virus isolates

#### Ferret-based map

**Fig. 1:**
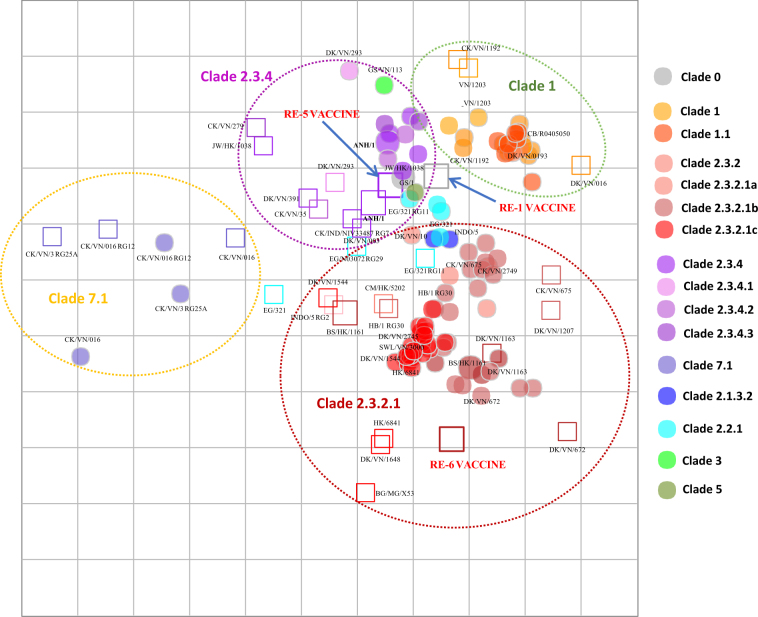
Antigenic maps of influenza A(H5N1) HA based on HI immune responses to chicken antisera. Both vertical and horizontal axes represent antigenic distance. Grid lines represent 1 antigenic distance unit, corresponding to a 2-fold dilution in the HI assay. Different antigenic clusters are indicated with different colors. Filled circles indicate antigens, while open squares indicate antisera

The antigenic distances between viruses based on HI analyses using ferret antisera are displayed in Fig. 1. Viruses within clades 1, 2.3.2.1, 2.3.4 and 7.1 were greatly inhibited by antisera raised in ferrets against viruses from the same clade, indicating close antigenic relationships within each clade (Fig. 1). Clade 1 viruses reacted well against clade 1 heterologous antisera within a 2- or 4-fold antigenic distance in ferret-based maps. Clade 2.3.2.1 viruses in the ferret-based map are clustered relatively closely, reflecting the high cross-reactivity among viruses of this group. This clade produced heterologous titers within two-fold of viruses among this subclade, but exhibited lower reactivity to more genetically distant clades. Clade 2.3.4 viruses reacted with patterns indicative of the genetic diversity found among viruses in this group. Although viruses clustered with those of the same subclade, among the various 2.3.4 subclades (i.e., 2.3.4.1, 2.3.4.2, and 2.3.4.3), there were greater antigenic distances (Fig. 1). The antisera produced against clade 2.3.4 viruses also reacted with high specificity for related viruses, although some clade 1 viruses were an exception (Fig. 1). Antisera against clade 7.1 viruses cross-reacted within this clade, but these viruses were outliers compared to all the other clades.

The use of ferret antisera for one-way analyses of additional test antigens yielded heterologous HI titers of sera against related and unrelated HPAI A(H5N1) within clade viruses, ranging from equivalent to 64-fold titer differences compared to homologous titers. From the ferret antisera-based map (Fig. 1), we observed that the majority of clade 2.3.2.1 (86%, *n* = 12/14) and clade 7.1 (67%, *n* = 2/3) viruses clustered closely with heterologous titers, within two-fold of homologous titers of genetically related viruses. Clade 2.3.4 and 1 viruses clustered close to each other, although variation was still observed within each clade and subclade. Eight of nine clade 2.3.4 viruses in the ferret antisera-based map were inhibited by antisera against other clade 2.3.4 viruses at titers within 4-fold of each other.

#### Chicken-based map

**Fig. 2:**
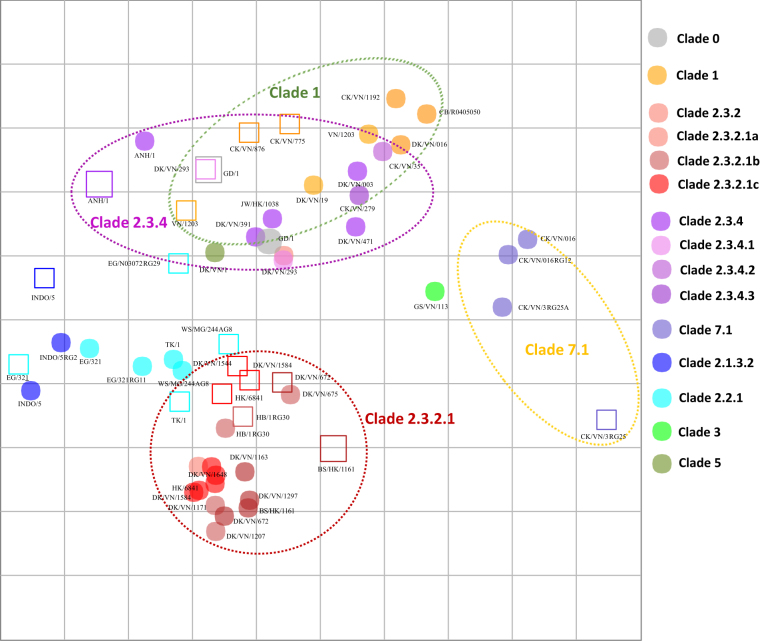
Antigenic maps of influenza A(H5N1) HA based on HI immune responses to ferret antisera. Both vertical and horizontal axes represent antigenic distance. Grid lines represent 1 antigenic distance unit, corresponding to a 2-fold dilution in the HI assay. Different antigenic clusters are indicated with different colors. Filled circles indicate antigens, while open squares indicate antisera

Two-way analyses of chicken antisera and the chicken antisera-based map (Fig. 2; Supplemental Table 2) showed a similar trend of within-clade clustering as that observed in the ferret antisera-based map (Fig. 1) for clades 1, 2.3.2.1, 2.3.4 and 7.1. The clade 1 viruses displayed shorter intra-clade antigenic distances (4-fold differences) than HI tests with ferret antisera (8-fold distances), which may be explained by the inclusion of additional clade 1.1.1 and 1.1.2 antigens in chicken HI tests. Similarly, the majority (73%, *n* = 8/11) of clade 2.3.2.1 viruses mapped within 4-fold of each other, while some viruses (27%, *n* = 3/11) showed 8-fold reduced titers relative to other viruses within clade 2.3.2.1. Clade 2.3.4 viruses showed strong intra-clade clustering (6 of 8 viruses were within 4-fold of clade 2.3.4 antisera), whereas two clade 2.3.4 antisera had 8-fold reduced titers relative to other clade 2.3.4 viruses (Fig. 2). Clustering of clade 2.3.2.1b viruses was less evident than for viruses of clades 2.3.2.1a and 2.3.2.1c. The estimated coordinates of the Re-6 vaccine virus (based on the nearest homologous strain of 2.3.2.1b) exhibited average geometric mean titers with a 5-fold difference to other viruses within the subclade. HPAI A(H5N1) viruses included on the map that have never circulated in Vietnam (i.e., the Egyptian clade 2.2.1 and Indonesian clade 2.1 viruses) and were not inhibited by heterologous antisera (i.e., sera from outside the clade) and vice versa. Thus, the clade 2.2.1 and 2.1 viruses did not show a consistent pattern of clustering across the chicken and ferret maps.

The Re-1 vaccine antigen (clade 0) was greatly inhibited by antisera raised against both clade 1 and 2.3.4 viruses, and the antisera raised with the Re-1 vaccine showed a reciprocal inhibition of both clade 1 and 2.3.4 antigens. In contrast, antisera against Re-1 showed little-to-no inhibition of clade 2.3.2.1 and 7.1 viruses. The Re-5 antisera (clade 2.3.4) reacted well against clade 1 and clade 2.3.4 antigens, whereas Re-6 antisera (clade 2.3.2.1b) antiserum reacted well with only the clade 2.3.2.1 antigens. Clade 7.1 viruses demonstrated relatively close antigenic relationships to one another but were outliers compared to all other clades; the three data points on the chicken antisera-based map demonstrated relatively close antigenic relationships to one another (Fig. 2).

For the one-way HI analyses (Supplemental Table 2), comparable titer profiles were observed for all members of clade 1, 1.1.1 and 1.1.2 viruses. Similarly, the thirty-nine test antigens from clade 2.3.2.1 viruses (comprising 10 clade 2.3.2.1a, 7 clade 2.3.2.1b and 22 clade 2.3.2.1c viruses), demonstrated moderate reactivity with most of within-clade heterologous antisera (64%, *n* = 7/11), with titers that were within 4-fold of each other. In contrast, the antigens had no reactivity to clade 1, 2.3.4 and 7 viruses. The clade 2.3.2.1a viruses separated into two antigenic clusters, whereas the clade 2.3.2.1c viruses overlapped within 2-fold of each other on the chicken antisera-based antigenic map and also clustered with some clade 2.3.2.1a viruses (Fig. 2). The clade 2.3.2.1b viruses were antigenically distinct from other clade 2.3.2.1 viruses. Similar to observations made in the two-way analyses, clade 2.3.2.1a and 2.3.2.1b viruses clustered together within 4-fold of one another but had greater antigenic distances compared to clade 2.3.2.1c viruses (within 2-fold). Clade 7.2 viruses had no reactivity to the clade 7.1 antisera tested. Although the clade 2.3.2.1b A/duck/Vietnam/NCVD-672/2011 virus reacted well with the genetically related clade 2.3.2.1b viruses, it was an outlier with respect to the other viruses of this clade, as it did not react well with antisera raised against clade 2.3.2.1a or 2.3.2.1c viruses (with 8- to 64-fold reductions observed compared to heterologous HI titers of clade 2.3.2.1a and 2.3.2.1c antisera). These relationships can be observed from the HI data matrices as well as the chicken antisera-based antigenic map (Fig. 2).

Similar to the results of the two-way HI titer comparison, vaccine antisera raised against Re-1 and Re-5 showed inhibition of their respective clade 1 and 2.3.4 antigens. Re-6 antiserum showed specific reactivity with only the clade 2.3.2.1 viruses. Clade 7.1 viruses were antigenically related to each other and remained outliers compared to all other clades. For viruses that have never circulated in Vietnam (i.e., clade 2.2, 2.2.1, 2.1.3), little reactivity to Vietnamese clade viruses was observed. In general, the results of the two-way and one-way analyses generated from HI tests using chicken antiserum panels were compatible to those produced with ferret antisera.

#### Combined chicken and ferret antisera-based map

**Fig. 3:**
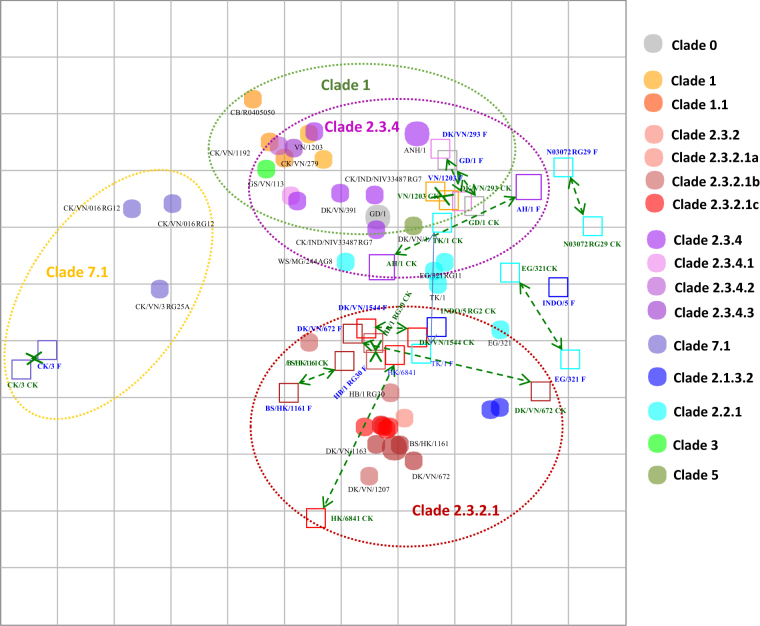
Map showing inferred relationships (antigenic distance units) between pairs of chicken and ferret reference antisera raised against the same viruses. Grid lines represent 1 antigenic distance unit, corresponding to a 2-fold dilution in the HI assay. Different antigenic clusters are indicated with different colors. Filled circles indicate antigens, while open squares indicate antisera. Green arrows with dotted lines indicate chicken and ferret antisera pairs

In general, the homologous HI titers indicated that four of fourteen pairs (28%) of chicken/ferret antisera had identical homologous HI titers (with titers ranging from 1:320 to 1:1280); eight pairs (57%) had higher HI titers in ferrets than in chickens, ranging from 2- to 8-fold higher (2 pairs had a 2-fold difference, 2 pairs had a 4-fold difference and 4 pairs had 8-fold difference); and 2 pairs (14%) had higher HI titers in chickens compared to ferrets (1 pair had a 2-fold difference and 1 pair had 4-fold difference). Side by side comparisons of chicken and ferret antisera run in the same HI test are shown in Fig. 3. The majority of antiserum pairs mapped to within proximate locations of each other (71%, *n* = 10/14). Antiserum pairs for clades 7.1 (*n* = 1), 1 (*n* = 1) and 0 (*n* = 1) showed nearly identical positions, as did most pairs in clades 2.3.2.1 (*n* = 3/5) and 2.3.4 (*n* = 1/2). However, three antigens that had homologous HI titers within 4- to 8-fold between chicken and ferret antisera pairs displayed greater fold reductions in side by side comparisons of heterologous titers generated from one-way testing of viruses. For instance, the clade 2.3.2.1 A/Hong Kong/6841/2010 (HK/6841) antigen had identical homologous titers to the chicken/ferret antiserum pair. However, the ferret antiserum displayed broad reactivity against unrelated clade viruses, whereas the chicken antiserum did not. Similarly, the clade 2.3.2.1b, A/duck/Vietnam/NCVD-672/2011 antiserum pairs had homologous HI titers within 4-fold of each other, while heterologous titers with non-clade 2.3.2.1 viruses varied from 8- to 32-fold, as expected. In particular, the ferret antiserum was able to inhibit clade 2.3.4 viruses, whereas chicken antisera did not. Together, these results suggest that this virus did not produce clade-specific antisera in either the chicken or ferret model. The clade 2.3.4 A/duck/Vietnam/NCVD-293/2009 virus also resulted in greater fold reductions (between 8- and 32-fold differences) in HI titers when comparing the reactivity of chicken and ferret antisera. Further analysis of the HI table indicated that 90% of all antigens (*n* = 38/42) were broadly inhibited by the A/duck/Vietnam/NCVD-293/2009 chicken antiserum, thus confounding the relative distance measures to different strains. Because the ferret HI included only two clade 2.3.4 viruses compared with the eight clade 2.3.4 viruses used in the chicken-based map, a direct comparison between chicken sera and ferret sera was not possible for these viruses. Another virus, clade 2.1.3.2 A/Indonesia/5/2005, also displayed greater fold reductions (between 8- and 32-fold differences) when comparing the reactivity of the homologous chicken and ferret antisera. The HI table shows that this virus produced a very high homologous HI titer of 1:1280 in the ferret, whereas the immune response in the chicken produced a titer of 1:160 (8-fold difference). This could be an anomaly in the ferret antiserum produced against this virus, suggesting that careful evaluation of initial homologous titers is warranted prior to use in routine HI testing.

#### Antigenic distances vs. vaccine efficacy in chickens

**Fig. 4:**
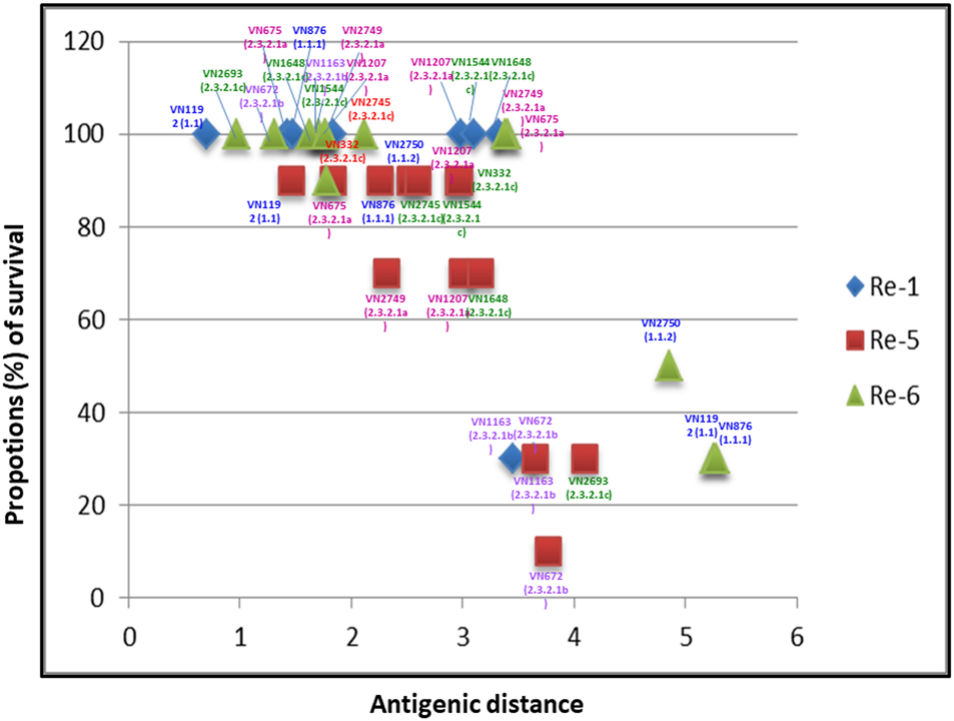
Antigenic distances between vaccine and challenge viruses plotted against vaccine efficacy (percent survival), as determined by in vivo vaccine challenge experiments. Each data point represents results from a challenge trial using 10 chickens/challenge virus and survival following intranasal inoculation with a dose of 10^6^ TCID_50_. Blue diamonds, red squares and green triangles represent chickens vaccinated with Re-1, Re-5 and Re-6, respectively. The challenge viruses used are identified for each group of vaccinated chickens with color-coded text indicating strain name and clade

For both ferret- and chicken-based antigenic analyses, the operative question posed by these studies is the degree to which the measured antigenic distances are useful for predicting the protective efficacy of different vaccine formulations. In vivo poultry vaccine challenge studies are performed by the Vietnamese Department of Animal Health on an annual or biannual basis to examine the protection afforded by poultry vaccines against viruses belonging to selected A(H5) clades and/or antigenic variants^6–9^. The compiled results of such studies are shown in Fig. 4, where percent survival is plotted against the ‘cartographic’ antigenic distance between a vaccine and the challenge virus. These studies have demonstrated that Re-1 (clade 0)-vaccinated chickens were protected against death when challenged with either clade 1 or clade 2.3.2.1a and 2.3.2.1c viruses (100% survival in all cases), whereas Re-1 was less effective against two clade 2.3.2.1b viruses (30%, *n* = 3/10) (Fig. 4). The clade 1 challenge viruses had antigenic distances to Re-1 that were ≤1.4 HAUs. The antigenic distance between the Re-1 vaccine strain and the clade 2.3.2.b viruses was ≥3.5 HAU, whereas the antigenic distances of other clade 2.3.2.1 challenge viruses were less. Similarly, the Re-5 (clade 2.3.4) vaccine provided good protection against clade 1 viruses (90%, *n* = 90/10) but less protection against clade 2.3.2.1 viruses (70–90%, *n* = 7–9/10 against clade 2.3.2.1a; 10–30%, *n* = 1–3/10 against clade 2.3.2.1b; and 30–90%, *n* = 3–9/10 against clade 2.3.2.1c viruses). Re-5 challenge viruses, resulting in 30% or less survival, had antigenic distances to Re-5 between 3.7–4.1 HAUs, while those with 70–90% survival had distances ranging from 1.5–3.2 HAUs (Fig. 4). The Re-6 (clade 2.3.2.1b) vaccine protected chickens against clade 2.3.2.1a, b and c viruses (90–100%, 9–10/10), but was less effective (30–50%, *n* = 3–5/10) against clade 1 viruses. The clade 1 viruses tested had antigenic distances of >4.9 HAUs, whereas all other challenge viruses were antigenically closer to the Re-6 vaccine strain (Fig. 4).

## Discussion

Vietnam is one of four countries (along with China, Egypt and Indonesia) where poultry vaccination has become a routine practice to control HPAI A(H5) viruses. To date, most government-supported poultry vaccination programs in Vietnam have employed inactivated, oil-in-water emulsion vaccines imported from the Harbin Veterinary Research Institute in China, which have been periodically updated and reformulated in an attempt to optimize antigenic matching following shifts in predominant H5 clade circulation in China. Phylogenetic analyses of codon-complete genome sequences of A(H5) viruses isolated from poultry outbreaks in Vietnam have identified a total of 56 genotypes and a circulation of at least 16 distinct clades^10–16^. From 2012 to 2015, six clades (1.1.2, 2.3.2.1a-c, 2.3.4.4 and 7.2) circulated, and the most recent surveillance data showed that clades 2.3.2.1c and 2.3.4.4 viruses predominated in 2015 and 2016^13^. Given the rapid evolution and frequent introduction of new A(H5) clades, maintaining a comprehensive panel of reference ferret antisera for antigenic characterization of viruses is crucial to assess antigenic matching of existing poultry vaccines. However, despite the importance of these reagents for successful vaccine strain selection and implementation policies, production of these panels remains resource-intensive and expensive to generate. To explore the utility of chicken antisera as a cost-effective strategy to meet these research goals, we ran HI assays using both chicken and ferret antisera and compared their ability to discriminate between antigenically distinct viruses using antigenic cartography.

Our results indicated that antigenic distances calculated using multidimensional scaling or ‘mapping’ approaches generated similar results for both the chicken and ferret HI data sets. In both instances, antigenic clustering corresponded to genetic grouping as determined by HA phylogenies. Clade 2.3.2.1 viruses were inhibited by antisera raised against clade 2.3.2.1 viruses at titers within 4-fold of each other but exhibited little or no cross-reactivity to clade 1, 7 and 2.3.4 viruses. Cross-HI tests of 2.3.2.1 viruses yielded heterologous titers within two-fold of homologous titers of genetically related viruses; these HI results translated into average intra-clade antigenic distance measures of 2.13 antigenic distance unit (ADU) (ranging from 0.15 to 4.95) on the antigenic map, vs. inter-clade differences of 3.84 ADU (ranging from 0.53 to 9.01). While clade 2.3.2.1c viruses exhibited a high degree of homogeneity in antigenic profiles (both serum and antigen points were tightly clustered), the 2.3.2.1a and 2.3.2.1b clades were not consistently inhibited by 2.3.2.1c antisera and vice versa (Fig. 1). Clade 1 viruses clustered together with titers within 4-fold of each other. Cross-HI tests of chicken antisera raised against clade 2.3.4 viruses showed strong intra-clade clustering (6 of 8 viruses were within 4-fold of clade 2.3.4 antisera), except for two clade 2.3.4 antisera that had 8-fold reduced titers against the clade 2.3.4 viruses.

Although analyses of the chicken and ferret antisera showed consistent reactivity patterns among tested the viruses and yielded similar comparisons of antigenic groupings, differences in reactivity and relative map positions were observed for 4 of the 14 chicken and ferret antisera pairs. For example, ferret antisera were unable to resolve antigenic differences between clade 1 and 2.3.4 viruses, which had overlapping antigenic space compared to the clearly defined delineation between these two clades using chicken antisera (Fig. 1, Fig. 2). On the other hand, clade 2.3.2.1 viruses clustered well in antigenic maps from ferret HI tests (within 1 antigenic unit) but were more widely dispersed in tests performed with chicken antisera. Variation in reactivity profiles of chicken vs. ferret antisera may be attributable to differences in antisera production. First, the production of ferret antisera, involved the inoculation of ferrets with live virus, followed by an adjuvanted boost 14 days post-infection. In contrast, the production of antisera in chickens required that inactivated virus be used for inoculation, as HPAI viruses cause fatal infections in chickens, which would have abrogated the possibility of collecting influenza-specific serum antibodies. Ferrets, on the other hand, experienced infections with the wild-type viruses that likely triggered both innate and cell-mediated immunity and stimulated pattern recognition receptors in a manner similar to natural infection. The production of reference anti-sera in chickens requires that they be immunized with an inactivated antigen due to the extreme lethality of HPAI A(H5) infections in chickens. Thus, the immune response measured by our chicken-based maps more closely mimicked vaccine-induced immunity rather than natural infection. These differences may be expected to impact the overall antibody titer but not the specificity of the serum antibodies. Although live virus infections may stimulate a broader, more robust immune response in chickens and reflect a natural infection, its practical application for generating antisera may be less advantageous. Utilization of a live virus requires that attenuated, reassortant/reverse genetics viruses are available because of the lethality of HPAI A(H5) viruses in chickens. In resource-limited situations, genetically modified, attenuated strains are expensive and time-consuming to produce, whereas inactivated virus is easily obtained from field strains. Furthermore, because poultry vaccines used in Vietnam rely on inactivated vaccine formulations, the generation of antisera with inactivated virus may be a better model of the post-vaccination immune response and better reflect the immune response to vaccination in the field. A direct comparison of antisera raised in ferrets using inactivated virus as an immunogen was not possible due, in part, to existing protocols approved by the Institutional Animal Care and Use Committee of the CDC that require the use of infectious virus but, more importantly, because previous studies showed that inactivated virus did not consistently elicit sufficient HI antibody titers when given intranasally (data not shown). A second important methodological difference was the use of different adjuvants for the ferrets and chickens. Adjuvants are compounds that have been shown to non-specifically augment host responses^17^. The precise mechanisms and differences between adjuvanted and non-adjuvanted responses have rarely been defined, and the impact they may have on the breadth of antibody coverage or specificity of the response is rarely measured with precision. For the experiments reported in this study using ferret antisera, Titermax Gold adjuvant was used in the boost inoculum, while the adjuvant used to produce reference chicken antisera was Montanide^TM^ ISA 70 VG. Both TiterMax Gold (http://www.titermax.com/technical-information.html) and Montanide^TM^ ISA 70 VG have been developed to create stable water-in-oil (W/O) emulsions. Although both adjuvants are metabolized in a similar fashion, it has not been assessed whether their use results in differences in antigenic cross-reactivity of serum antibodies. Finally, besides the methodological differences noted, there is likely to be some variation in how the ferret immune system targets viral epitopes compared to that of chickens. Host-specific variation in antigen processing, antibody production, and ultimately, the specificity of an antibody for an antigen may lead to variation in HI reactivity patterns between antisera raised in each host.

Plotting of the antigenic distance relative to percent survival of chickens post-vaccine challenge demonstrated an inverse relationship between survival rate and antigenic distance. In general, the comparison of antigenic distances and percent survival of chickens following vaccine challenge suggested a trend toward lower survival rates as the antigenic distance between a challenge virus and vaccine virus increased. Indeed, aggregate results of in vivo challenge studies showed that Re-1 (clade 0) reliably protected chickens against clinical disease when challenged with either clade 1 or clade 2.3.2.1a and c viruses, but protection against clade 2.3.2.1b viruses was reduced when antigenic distances exceeded 3.5 units. The Re-5 (clade 2.3.4) vaccine provided good protection against clade 1 viruses but was more variable and less protective in experiments using the clade 2.3.2.1 a, b and c challenge viruses. Challenge viruses resulting in 30 to 10% survival had antigenic distances to the vaccine strain beyond 3.5 HAUs. The Re-6 (clade 2.3.2.1b) vaccine protected chickens against challenge with clade 2.3.2.1a, b and c viruses but only partially protected birds from clade 1 virus challenges. The infection of chickens with clade 1 viruses, which were the most antigenically distant to the vaccine strains in this study, resulted in poor protection. Additional studies assessing a larger number of challenge viruses and more recent poultry vaccines are warranted. The predicted protection profile of vaccines challenged with clade 2.3.4.4 viruses, which have spread into North America and continue to spread in Europe, Africa and Asia, is of particular interest, as this was the predominant clade circulating in Vietnam in 2016, as evidenced by the extensive national active surveillance system in live bird markets (LBM)^13^. Additional HI test data with these viruses is needed to further assess the correlation between antigenic distances and the in vivo protection of poultry vaccines when chickens are challenged with clade 2.3.4.4 viruses. The antigenic data presented in this study supports current recommendations for use of the Re-6 (2.3.2.1b) vaccine for outbreaks of clade 2.3.2.1c viruses, but additional data are needed to assess circulating strains of clade 2.3.4.4 viruses. A new bivalent vaccine formulation, Re-8 (derived from clade 2.3.4.4 A(H5N6) A/chicken/Guizhou/4/2013 mixed with Re-6), is currently under evaluation and shows promise for generating broader protection against antigenically diverse viruses. What remains to be seen is whether the precise quantitative measures of antigenic distances, as afforded by the cartographic analyses presented here, could be used to model and predict outcomes from experimental challenge studies. For example, it should be feasible to examine, compare, and visualize how the breadth of immunity induced by bivalent vaccine formulations (i.e., comprising two A(H5) clade variants) differs from immune responses to monovalent formulations through an antibody landscaping approach that builds upon the A(H5) maps presented here.

In summary, our data demonstrate the utility of using panels of reference chicken antisera for the systematic analysis of antigenic drift variants of A(H5) viruses and indicates that the chicken- and ferret-based antiserum panels used in HI assays yielded comparable antigenic profiles, which were relevant for evaluating A(H5) vaccine antigenic matches. The great advantage of using antisera raised in chickens for these analyses is the feasibility of generating the antigenic data quickly *in-country* and within the existing constraints and resources of national animal health laboratories in Vietnam. We strongly advocate for more integrated analyses of antigenic distance measures based on cartography, together with output from in vivo transmission studies, to develop reliable surrogate markers of protective immunity and standardized measures of vaccine performance. Strategic use of new computational tools is needed to meet the long term goal of achieving robust vaccine-induced heterosubtypic immunity and to overcome the Achilles’ heel of antigenic drift.

## Materials and methods

### Viruses

A total of 85 Vietnamese HPAI A(H5N1) viruses were selected to represent the historical and contemporary diversity of hemagglutinin (HA) clades/subclades from 2001-2014^18^. Fifteen additional non-Vietnamese A(H5) viruses were also included, representing ancestral (prototype) clades and viruses from Indonesia and Egypt that have not circulated in Vietnam. In total, the panel of viruses represented 21 out of 22 existing A(H5) clades. The poultry vaccines evaluated in this study were produced by the Harbin Veterinary Research Institute in Harbin, China, consisting of inactivated reverse engineered vaccine constructs that were generated using the A/Puerto Rico/8/1934 (PR8) backbone and the HA and NA genes from various A(H5) clades. The vaccines of interest were Re-1 (A/goose/Guangdong/1/1996, clade 0), Re-5 (A/duck/Anhui/1/2006, clade 2.3.4), Re-6 (A/duck/Guangdong/S1322/2006, clade 2.3.2.1b) and Re-4 (A/chicken/Shanxi/2/2006, clade 7.1). As we did not have access to the vaccine viruses for Re-4, Re-5, or Re-6, the following closely related viruses were used as surrogates (>99% identical HA nucleotide sequences): Re-5 (A/Anhui/1/2005, clade 2.3.4), Re-4 (A/chicken/Vietnam/NCVD-016/2008 IDCDC-RG12, clade 7.1) and Re-6 (A/barn-swallow/Hong Kong/D10-1161/2010, clade 2.3.2.1b).

### Antigen preparation

Viruses were grown in 9- or 10-day-old influenza-free, clean-embryonated chicken eggs (C-ECE). Ten C-ECEs per isolate were used, and the allantoic fluid was harvested from each C-ECE after incubation at 37 °C for 48–72 h. After harvesting, viruses were inactivated with beta-propiolactone (BPL) at a final concentration of 0.05% for 4 h at 37 °C, after which the virus samples were stored at 4 °C overnight and then adjusted to pH 7.2 using a sterile 7.5% NaHCO_3_ solution. Inactivation was confirmed through three serial egg passages without any evidence of viral growth as detected by HA. Inocula for chicken antisera production were prepared by mixing inactivated antigens [at 1280 hemagglutination units (HAU)] with an adjuvant (Montanide ISA 70) in a 3:7 v:v ratio, which were emulsified by sonication for 15–20 s as per manufacturer’s instructions (http://www.seppic.com/animal-health/vaccine-adjuvant/montanide-isa/-montanide-isa-70-vg). The emulsified antigen-adjuvant mixtures were stored at 4 °C and used within 24–48 h of preparation.

### Chicken antisera production

Chickens were obtained as day-old chicks from a commercial hatchery that was certified as being free of avian influenza viruses by the Department of Animal Health (DAH) in Hanoi, Vietnam. The antibody-negative status of chickens was reconfirmed at 5 weeks of age, prior to being used for antisera production, by testing with a commercial NP ELISA Flock Check AI (Idexx Laboratories, Westbrook, ME, USA). Five chickens were used per viral antigen for antisera production. Briefly, chickens were immunized subcutaneously in the nape of the neck with 0.5 ml of immunogen/adjuvant using a 5-ml syringe and a 22 gauge needle. To produce antisera against commercial poultry vaccines, vaccines were reconstituted as per manufacturer’s instructions (Harbin Veterinary Research Institute, Harbin, China). At 3 weeks post-immunization, chickens were sedated by intravenous administration of ketamine/xylazine based on body weight, and blood was collected by cardiac puncture. Whole blood tubes were incubated for 3–5 h at 37 °C on their sides, after which they were placed at 4 °C overnight to maximize serum volume. Sera were harvested by centrifuging the samples to pellet the clots, after which the supernatants were decanted and stored at −20 °C in 1-ml aliquots until further use. Procedures for animal work at the NCVD were adapted from the OIE (available at: http://web.oie.int/eng/normes/mcode/en_chapitre_1.7.8.htm).

### Ferret antisera production

Ferret antisera against the selected A(H5) viruses were produced as previously described at the Centers for Disease Control and Prevention (CDC) Influenza Division in Atlanta, GA, USA. Briefly, outbred male ferrets with body weights ranging from 900 to 1800 g and less than one year of age were obtained from Triple F Farms (Sayre, PA, USA). Ferrets were confirmed as being antibody-negative for influenza A and B viruses and were inoculated intranasally (in) with doses ranging between 10^4^ and 10^6^ EID_50_ of live virus diluted in PBS. At 14 days post-inoculation, each ferret was boosted by subcutaneous injection in both hind legs with at least 1024 HAU of virus mixed with Titermax Gold Adjuvant (Sigma-Aldrich, MO, USA). Antisera were collected 14 days post-boost. All work with animals was carried out in biosafety level 3 laboratories with enhancements to meet USDA/APHIS guidelines and performed under protocols approved by the Institutional Animal Care and Use Committee of the CDC.

### Comparison of chicken and ferret antisera via HI assays

Antigenic characterization was performed using the post-infection ferret and chicken antisera in parallel. Sera were heat inactivated at 56 °C for 30 min, absorbed with packed chicken red blood cells (CRBCs) to remove non-specific agglutinators, and then tested through an HI assay with 0.5% CRBCs following standard procedures^19^. Starting dilutions of 1:10 were used, and endpoint titers were calculated as the reciprocal of the last HI positive serum dilution. Naive, negative control sera from ferrets and chickens were used in all HI tests to control for potential background reactivity (data not shown).

### Antigenic cartography

Antigenic cartography was performed as previously described^20^ using the open access software available through https://acmacs-web.antigenic-cartography.org/. HI tables containing the full panel of HI titers (homologous and heterologous) were first normalized by calculating the difference between the log2 (HI titer) of a given virus-serum pair and the maximum log2 (HI titer) of that serum against a given virus. Antigenic distances were calculated using multidimensional scaling to minimize the differences between the target distances and the distances in the antigenic map. Five-hundred dimensional annealing runs were performed for optimization, with random restarts implemented to avoid being trapped in local optima. Metadata associated with all reference and test antigens were prepared in Microsoft Office Excel 2010 spreadsheets with an antigen table (test virus, antigen ID, strain name, clade, and abbreviation) and antisera table (test serum, serum ID, serum strain, serum clade, abbreviation, and serum type). Antigenic maps were visualized using Tableau Reader v9.1 by importing x/y coordinate output files and linking each coordinate with reference or test antigen metadata.

### HI data matrices

A total of 18 ferret antisera were used for cross-HI tests with 15 homologous antigens and 25 test antigens. Similarly, the 29 chicken antisera experimentally produced in this study were used for cross-HI testing with their homologous antigens and 65 heterologous test antigens (circulating field viruses) (Table 2). Post-vaccination sera of Re-1 (A/goose/Guangdong/1/1996, clade 0), Re-5 (A/duck/Anhui/1/2006, clade 2.3.4), and Re-6 (A/duck/Guangdong/S1322/2006, clade 2.3.2.1b) vaccines were titrated against the full complement of antigens (29 reference viruses and 65 test antigens). For 14 viruses, we had access to both ferret and chicken antisera raised against the same virus; these sera were titrated against the 14 homologous antigens and 28 test antigens.

**Table 2:** Viruses used in this study

	Isolate name	Abbreviation	Viral status	Clade	Antigen type
1	A/goose/Guangdong/1/1996	GD/1/96	WT	0	Reference
2	A/Vietnam/1203/2004	VN/1203/04	WT	1	Reference
3	A/duck/Vietnam/NCVD-016/2007	DK/VN/016/07	WT	1.1	Reference^a^
4	A/Cambodia/R0405050/2007	CB/R0405050/07	WT	1.1	Reference
5	A/chicken/Vietnam/NCVD-1192/2012	CK/VN/1192/12	WT	1.1	Reference^a^
6	A/Indonesia/5/2005	INDO/5/05	WT	2.1.3.2	Test
7	A/Indonesia/5/2005 (CDC-RG2)	INDO/5/05 RG2	RG	2.1.3.2	Reference
8	A/turkey/Turkey/1/2005	TK/1/05	WT	2.2.1	Reference
9	A/Egypt/321-Namru3/2007	EG/321/07	WT	2.2.1	Reference
10	A/Egypt/321-Namru3/2007 (IDCDC-RG11)	EG/321/07 RG11	RG	2.2.1	Reference^a^
11	A/Egypt/N03072/2010 (IDCDC-RG29)	EG/N03072/10 RG29	RG	2.2.1	Reference
12	A/common magpie/Hong Kong/5052/2007	CM/HK/5052/07	WT	2.3.2.1	Reference^a^
13	A/Hubei/1/2010 (IDCDC-RG30)	HB/1/10 RG30	RG	2.3.2.1a	Reference
14	A/chicken/Vietnam/NCVD-675/2011	CK/VN/675/11	WT	2.3.2.1a	Reference^a^
15	A/duck/Vietnam/NCVD-1207/2012	DK/VN/1207/12	WT	2.3.2.1a	Reference^a^
16	A/barn-swallow/Hong Kong/D10-1161/2010	BS/HK/1161/10	WT	2.3.2.1b	Reference
17	A/duck/Vietnam/NCVD-672/2011	DK/VN/672/11	WT	2.3.2.1b	Reference
18	A/duck/Vietnam/NCVD-1163/2012	DK/VN/1163/12	WT	2.3.2.1b	Reference^a^
19	A/bar-headed goose/Mongolia/X53/2009	BG/MG/X53/09	WT	2.3.2.1c	Reference^a^
20	A/Hong Kong/6841/2010	HK/6841/10	WT	2.3.2.1c	Reference
21	A/duck/Vietnam/NCVD-1648/2012	DK/VN/1648/12	WT	2.3.2.1c	Reference^a^
22	A/duck/Vietnam/NCVD-1544/2012	DK/VN/1544/12	WT	2.3.2.1c	Reference
23	A/duck/Vietnam/NCVD-1584/2012	DK/VN/1584/12	WT	2.3.2.1c	Reference^b^
24	A/duck/Vietnam/NCVD-003/2008	DK/VN/003/08	WT	2.3.4	Reference^a^
25	A/duck/Vietnam/NCVD-391/2009	DK/VN/391/09	WT	2.3.4	Reference^a^
26	A/Japanese-white-eye/Hong Kong/1038/06	JW/HK/1038/06	WT	2.3.4	Reference^a^
27	A/chicken/India/NIV33487/2006 (IBCDC-RG7)	CK/IND/NIV33487/06 RG7	RG	2.3.4	Reference^a^
28	A/Anhui/1/2005	ANH/1/05	RG	2.3.4	Reference
29	A/duck/Vietnam/NCVD-293/2009	DK/VN/293/09	WT	2.3.4.1	Reference
30	A/chicken/Vietnam/NCVD-35/2008	CK/VN/35/08	WT	2.3.4.2	Reference^a^
31	A/chicken/Vietnam/NCVD-279/2009	CK/VN/279/09	WT	2.3.4.3	Reference^a^
32	A/chicken/Vietnam/NCVD-016/2008	CK/VN/016/08	WT	7.1	Reference^a^
33	A/chicken/Vietnam/NCVD-016/2008 (IDCDC-RG12)	CK/VN/016/08 RG12	RG	7.1	Reference^a^
34	A/chicken/Vietnam/NCVD-3/2008 (IDCDC-RG25A)	CK/VN/3/08 RG25A	RG	7.1	Reference
35	A/goose/Vietnam/113/2001	GS/VN/113/01	WT	3	Test
36	A/duck/Vietnam/NCVD-1/2002	DK/VN/1/02	WT	5	Test
37	A/whooper swan/Mongolia/244/2005 (Ag8)	WS/MG/244/05 AG8	WT	2.2	Reference^b^
38	A/duck/Vietnam/10/2005	DK/VN/10/05	WT	2.3.2	Test
39	A/duck/Vietnam/NCVD-19/2003	DK/VN/19/03	WT	1	Test
40	A/duck/Vietnam/NCVD-2153/2012	DK/VN/2153/12	WT	1.1.2	Test
41	A/chicken/Vietnam/NCVD-2750/2013	CK/VN/2750/13	WT	1.1.2	Test
42	A/chicken/Vietnam/NCVD-1096/2013	CK/VN/1096/13	WT	7.2	Test
43	A/chicken/Vietnam/NCVD-93/2008	CK/VN/93/08	WT	7.2	Test
44	A/chicken/Vietnam/NCVD-2749/2013	CK/VN/2749/13	WT	2.3.2.1a	Test
45	A/chicken/Vietnam/NCVD-2693/2013	CK/VN/2693/13	WT	2.3.2.1b	Test
46	A/duck/Vietnam/NCVD-2745/2013	DK/VN/2745/13	WT	2.3.2.1c	Test
47	A/chicken/Vietnam/NCVD-14-A318/2014	CK/VN/A318/14	WT	2.3.2.1c	Test
48	A/duck/Vietnam/NCVD-14-A332/2014	DK/VN/A332/14	WT	2.3.2.1c	Test

Antigens were produced in embryonating chicken eggs for all strains. Chicken antisera were produced for strains and were designated as ‘reference’ antigens

*WT* wild type, *RG* viruses produced by reverse genetics with PR8 internal genes and with the polybasic cleavage site removed from the HA protein

^a^Reference antigen for the chicken model only

^b^Reference antigen for the ferret model only

### Rationale of the HI test set-up

Following the conventions and terminology of antigenic characterizations of serological data sets, we refer to ‘two-way’ vs. ‘one-way’ analyses of HI titer matrices. Two-way analyses refer to interpretations of antigenic relationships based on complete data sets, where each virus is represented by both an antigen and its homologous antiserum. For two-way analyses, the spatial coordinates for a given antigen or antiserum reflect the differential reactivity to both homologous and heterologous viruses. For the purposes of comparing chicken vs. ferret models, we first performed two-way analyses to assess whether the chicken and ferret models generated antisera with comparable specificities. In contrast to ‘two-way’ analyses, a ‘one-way’ analysis involves interpretation of antigenic relationships when a given virus is represented solely by the viral antigen because no homologous antiserum has been produced (in this case the antigens are called ‘test’ antigens). For one-way analyses, the spatial coordinates for a given antigen are derived from the combined reactivity of a given antigen to a panel of heterologous antisera. The most common scenario for cartography is to have a small number of reference antigens and a much larger number of test antigens because antisera are expensive and time-consuming to generate.

### Plotting of antigenic distances to in vivo vaccine efficacy

Antigenic distances, calculated from chicken antisera-based HI tests, between viruses previously tested in vaccine challenge studies and each of three poultry vaccines (i.e., Re-1, Re-5, and Re-6) were calculated as above. The distances were plotted against the percent survival of chickens that were vaccinated and challenged with the corresponding vaccine and virus. The methodology and results from the vaccine challenge studies were previously described^6–9^.

### Chicken challenge studies

The commercial inactivated H5N1 Re-1, Re-5 and Re-6 poultry vaccines, which were previously and currently used in Vietnam and produced by the Harbin Veterinary Research Institute (People’s Republic of China), were used to vaccinate chickens. Chickens were obtained at one day of age from a commercial hatchery. Serum samples were collected from 20 chickens in each experiment to ascertain that the birds were serologically negative for antibodies to the NP protein of influenza A viruses as determined by the commercial ELISA test Flock Check AI (Idexx Laboratories, Westbrook, ME). Two-week-old chickens (20 chickens/group) were vaccinated subcutaneously in the nape of the neck with 0.5 ml of the Re-1, Re-5 or Re-6 vaccines. This vaccination schedule was based on the manufacturer’s recommendations. In each experiment, an additional 10 chickens were not vaccinated and served as the challenge controls.

Three weeks after vaccination, blood samples were collected and tested by hemagglutinin tests (HI) with inactivated antigens of A/VN/1203/2004 (for the Re-1 vaccinated chicken group), A/Anhui/1/2005 (for the Re-5 vaccinated chicken group), and A/Hubei/1/2010 (for the Re-6 vaccinated chicken group). Ten chickens with HI titers ≥ 3 log2 from each vaccinated group were selected for challenge experiments, which were inoculated intranasally with 10^6^ TCID_50_ of the selected H5 HPAI challenge virus. Chickens were observed daily for clinical signs and mortality. Oropharyngeal and cloacal swabs were collected at different days post challenge (dpc) to assess viral shedding. At the end of each experiment (10 days), blood was collected from all surviving chickens for antibody assays. All experiments were performed in biosafety level two plus enhanced facilities at the National Centre for Veterinary Diagnostics.

## Electronic supplementary material

Supplemental Figures 1

Supplemental Tables
