# Supplementary figures and images for: Antigenic characterization of highly pathogenic avian influenza A(H5N1) viruses with chicken and ferret antisera reveals clade-dependent variation in hemagglutination inhibition profiles
Source: Emerg Microbes Infect. 2018 May 31;7:100. doi: 10.1038/s41426-018-0100-7 (PMC5981457; doi:10.1038/s41426-018-0100-7)

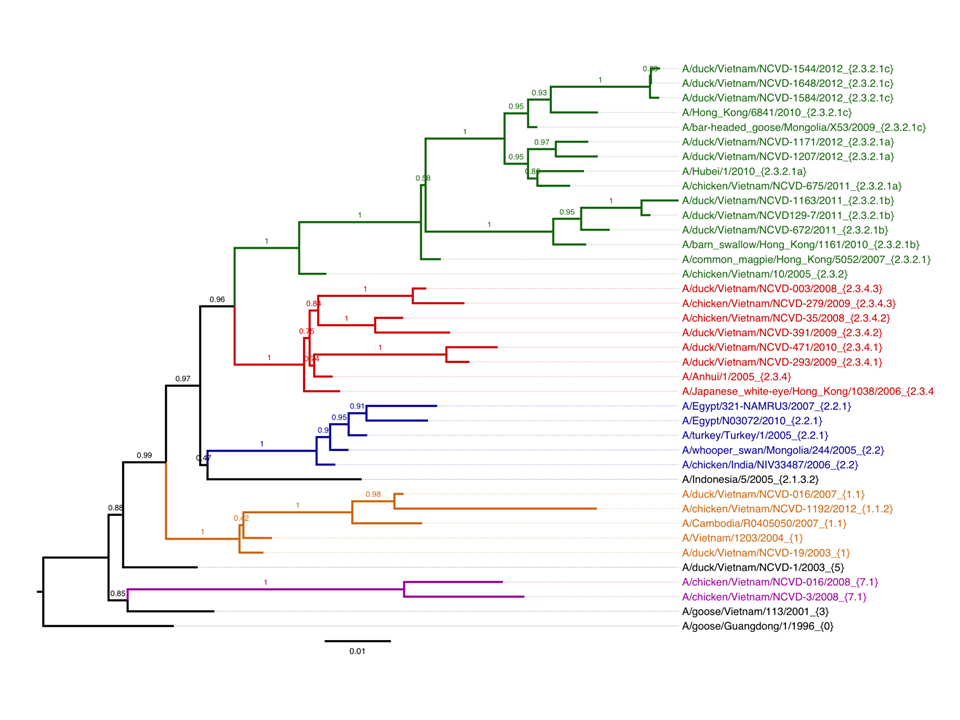

Supplement: Supplementary file 1 — Supplemental Figures 1 [file 41426_2018_100_MOESM1_ESM.tif]
