# Supplementary material for: Antigenic characterization of highly pathogenic avian influenza A(H5N1) viruses with chicken and ferret antisera reveals clade-dependent variation in hemagglutination inhibition profiles
Source: Emerg Microbes Infect. 2018 May 31;7:100. doi: 10.1038/s41426-018-0100-7 (PMC5981457; doi:10.1038/s41426-018-0100-7)
Supplement: Supplementary file 2 — Supplemental Tables [file 41426_2018_100_MOESM2_ESM.docx]

**Supplemental Table 1**: HI fold differences using chicken antisera

| **REFERENCE ANTIGENS** | | **Clade** | **VN/ 1203** | **VN/DK/016** | **CB/ R0405050** | **DK/VN/ 1192** | **IND/5/RG2** | **TK/1** | **EG/ 321** | **EG/321/RG11** | **N03072/2010 RG29** | **CM/HK/5052** | **HB/1/RG30** | **CK/VN/675** | **DK/VN/1207** | **BS/HK/1161** | **DK/VN/672** | **DK/VN/1163** | **BHG/MG/X53** | **HK/ 6841** | **CK/VN/ 1648** | **DK/VN/ 1544** | | **DK/VN/ 003** | | **DK/VN/ 391** | | **JWE/HK/ 1038** | | **CK/IND/ 33487/RG7** | | **ANH/1** | | **DK/VN/ 293** | | **CK/VN/ 35** | | **CK/VN/ 279** | | **CK/VN/ 016** | | **CK/VN/016/ RG12** | | **CK/VN/3/ RG25A** | |  |  |  |
| --- | --- | --- | --- | --- | --- | --- | --- | --- | --- | --- | --- | --- | --- | --- | --- | --- | --- | --- | --- | --- | --- | --- | --- | --- | --- | --- | --- | --- | --- | --- | --- | --- | --- | --- | --- | --- | --- | --- | --- | --- | --- | --- | --- | --- | --- | --- | --- | --- |
| 1 | A/goose/Guangdong/1/1996 | 0 | 1 | 4 | 4 | 1 | 4 | 2 | 4 | 2 | 4 | 2 | 2 | 4 | 1 | 2 | 16 | 4 | 64 | 32 | 16 | 1 | | 4 | | 2 | | 4 | | 4 | | 2 | | 0.25 | | 4 | | 0.5 | | 1 | | 32 | | 32 | |  |  |  |
| 2 | A/Vietnam/1203/2004 | 1 | 1 | 0.5 | 2 | 0.5 | 4 | 16 | 16 | 4 | 4 | 8 | 2 | 4 | 8 | 8 | 128 | 16 | 64 | 128 | 64 | 4 | | 8 | | 8 | | 64 | | 16 | | 2 | | 0.25 | | 32 | | 4 | | 16 | | 64 | | 128 | |  |  |  |
| 3 | A/duck/Vietnam/NCVD-016/2007 | 1.1 | 2 | 1 | 1 | 0.5 | 8 | 8 | 16 | 4 | 4 | 4 | 2 | 2 | 2 | 4 | 64 | 16 | 64 | 64 | 32 | 4 | | 8 | | 4 | | 16 | | 16 | | 8 | | 0.13 | | 8 | | 2 | | 8 | | 64 | | 128 | |  |  |  |
| 4 | A/Cambodia/R0405050/2007 | 1.1 | 4 | 1 | 1 | 16 | 16 | 16 | 16 | 8 | 8 | 4 | 2 | 16 | 8 | 16 | 64 | 16 | 128 | 128 | 32 | 32 | | 8 | | 8 | | 8 | | 16 | | 8 | | 2 | | 8 | | 8 | | 16 | | 64 | | 128 | |  |  |  |
| 5 | A/chicken/Vietnam//NCVD-1192/2012 | 1.1 | 4 | 1 | 1 | 1 | 8 | 8 | 16 | 4 | 8 | 4 | 2 | 2 | 4 | 4 | 64 | 16 | 64 | 64 | 32 | 8 | | 4 | | 4 | | 16 | | 8 | | 1 | | 0.5 | | 8 | | 4 | | 16 | | 64 | | 128 | |  |  |  |
| 6 | A/Indonesia/5/2005 | 2.1.3.2 | 8 | 4 | 1 | 8 | 1 | 8 | 8 | 2 | 2 | 1 | 2 | 16 | 4 | 2 | 4 | 8 | 32 | 16 | 32 | 4 | | 8 | | 8 | | 16 | | 8 | | 1 | | 8 | | 8 | | 1 | | 8 | | 64 | | 128 | |  |  |  |
| 7 | A/Indonesia/5/2005 (CDC-RG2) | 2.1.3.2 | 8 | 4 | 2 | 16 | 1 | 8 | 8 | 2 | 2 | 1 | 2 | 32 | 8 | 2 | 4 | 16 | 32 | 8 | 32 | 4 | | 4 | | 2 | | 16 | | 8 | | 4 | | 8 | | 4 | | 0.5 | | 16 | | 64 | | 128 | |  |  |  |
| 8 | A/turkey/Turkey/1/2005 | 2.2.1 | 2 | 2 | 8 | 4 | 4 | 1 | 1 | 1 | 1 | 1 | 1 | 8 | 0.25 | 2 | 8 | 4 | 64 | 64 | 4 | 2 | | 8 | | 4 | | 16 | | 2 | | 4 | | 0.25 | | 16 | | 2 | | 8 | | 64 | | 64 | |  |  |  |
| 9 | A/Egypt/321-Namru3/2007 | 2.2.1 | 4 | 2 | 1 | 4 | 2 | 2 | 1 | 1 | 2 | 1 | 2 | 16 | 2 | 4 | 4 | 8 | 128 | 16 | 16 | 8 | | 8 | | 4 | | 8 | | 4 | | 4 | | 0.5 | | 16 | | 2 | | 8 | | 64 | | 64 | |  |  |  |
| 10 | A/Egypt/321-Namru3/2007 (IDCDC-RG11) | 2.2.1 | 2 | 2 | 1 | 1 | 2 | 1 | 0.5 | 1 | 2 | 1 | 2 | 16 | 4 | 4 | 8 | 8 | 128 | 64 | 8 | 2 | | 4 | | 1 | | 16 | | 1 | | 2 | | 0.13 | | 8 | | 2 | | 8 | | 32 | | 128 | |  |  |  |
| 11 | A/Egypt/N03072/2010 (IDCDC-RG29) | 2.2.1 | 2 | 4 | 4 | 0.5 | 1 | 1 | 1 | 2 | 1 | 0.5 | 1 | 16 | 0.25 | 4 | 4 | 2 | 32 | 64 | 4 | 1 | | 1 | | 8 | | 8 | | 1 | | 1 | | 0.13 | | 32 | | 1 | | 8 | | 64 | | 128 | |  |  |  |
| 12 | A/Common magpie/Hongkong/5052/2007 | 2.3.2.1 | 16 | 4 | 8 | 16 | 2 | 32 | 2 | 1 | 2 | 1 | 1 | 2 | 2 | 1 | 8 | 8 | 64 | 16 | 8 | 4 | | 8 | | 8 | | 16 | | 16 | | 8 | | 0.25 | | 8 | | 4 | | 16 | | 64 | | 128 | |  |  |  |
| 13 | A/Hubei/1/2010 (IDCDC-RG30) | 2.3.2.1 a | 4 | 2 | 0.5 | 8 | 2 | 16 | 4 | 1 | 4 | 1 | 1 | 2 | 0.5 | 2 | 4 | 4 | 8 | 2 | 2 | 2 | | 8 | | 8 | | 64 | | 8 | | 2 | | 0.25 | | 2 | | 2 | | 16 | | 64 | | 128 | |  |  |  |
| 14 | A/chicken/Vietnam/NCVD-675/2011 | 2.3.2.1a | 4 | 8 | 4 | 2 | 2 | 32 | 16 | 8 | 4 | 1 | 1 | 1 | 1 | 2 | 8 | 4 | 16 | 16 | 8 | 8 | | 4 | | 4 | | 32 | | 8 | | 4 | | 0.5 | | 4 | | 1 | | 16 | | 64 | | 128 | |  |  |  |
| 15 | A/duck/Vietnam/NCVD-1207/2012 | 2.3.2.1a | 32 | 8 | 8 | 16 | 4 | 32 | 32 | 8 | 4 | 1 | 2 | 2 | 1 | 2 | 32 | 4 | 4 | 4 | 2 | 4 | | 8 | | 8 | | 64 | | 8 | | 4 | | 2 | | 4 | | 2 | | 16 | | 64 | | 128 | |  |  |  |
| 16 | A/barn Swallow/Hong Kong/D10-1161/2010 | 2.3.2.1 b | 64 | 8 | 8 | 16 | 2 | 16 | 4 | 2 | 8 | 1 | 1 | 4 | 1 | 1 | 2 | 2 | 16 | | | | 16 | | 4 | | 4 | | 8 | | 8 | | 64 | | 16 | | 16 | | 2 | | 16 | | 16 | | 16 | | 64 | 128 |
| 17 | A/duck/Vietnam/NCVD-672/2011 | 2.3.2.1b | 32 | 8 | 8 | 16 | 4 | 32 | 4 | 2 | 16 | 1 | 1 | 4 | 1 | 1 | 1 | 2 | 16 | 16 | 4 | 4 | | 8 | | 8 | | 64 | | 16 | | 16 | | 1 | | 32 | | 16 | | 16 | | 64 | | 128 | |  |  |  |
| 18 | A/duck/Vietnam/NCVD-1163/2012 | 2.3.2.1b | 32 | 8 | 8 | 16 | 4 | 32 | 4 | 1 | 16 | 1 | 2 | 4 | 0.5 | 2 | 8 | 1 | 32 | 16 | 8 | 4 | | 8 | | 16 | | 64 | | 16 | | 16 | | 2 | | 32 | | 16 | | 16 | | 64 | | 128 | |  |  |  |
| 19 | A/Barheaded goose/Mongolia/X53/2009 | 2.3.2.1c | 32 | 8 | 8 | 16 | 2 | 32 | 8 | 2 | 2 | 2 | 1 | 4 | 0.5 | 2 | 8 | 4 | 1 | 1 | 0.5 | 0.5 | | 8 | | 8 | | 16 | | 16 | | 4 | | 0.5 | | 4 | | 2 | | 16 | | 64 | | 128 | |  |  |  |
| 20 | A/Hongkong/6841/2010 | 2.3.2.1c | 32 | 8 | 8 | 32 | 2 | 32 | 4 | 8 | 2 | 1 | 1 | 2 | 1 | 2 | 16 | 4 | 4 | 1 | 1 | 0.5 | | 8 | | 8 | | 64 | | 16 | | 4 | | 0.5 | | 4 | | 4 | | 16 | | 64 | | 128 | |  |  |  |
| 21 | A/duck/Vietnam/NCVD-1648/2012 | 2.3.2.1c | 32 | 8 | 8 | 16 | 1 | 32 | 4 | 8 | 2 | 1 | 1 | 2 | 1 | 2 | 16 | 4 | 4 | 1 | 1 | 2 | | 8 | | 8 | | 64 | | 16 | | 4 | | 1 | | 8 | | 2 | | 16 | | 64 | | 128 | |  |  |  |
| 22 | A/duck/Vietnam/NCVD-1544/2012 | 2.3.2.1c | 16 | 8 | 8 | 8 | 1 | 32 | 4 | 4 | 2 | 2 | 1 | 2 | 0.5 | 1 | 16 | 2 | 2 | 1 | 1 | 1 | | 8 | | 8 | | 64 | | 16 | | 4 | | 0.5 | | 8 | | 2 | | 16 | | 64 | | 128 | |  |  |  |
| 23 | A/duck/Vietnam/NCVD-003/2008 | 2.3.4 | 4 | 8 | 8 | 8 | 8 | 8 | 8 | 4 | 8 | 4 | 8 | 8 | 8 | 8 | 32 | 16 | 128 | 64 | 64 | 16 | | 1 | | 2 | | 16 | | 4 | | 4 | | 2 | | 4 | | 1 | | 16 | | 64 | | 128 | |  |  |  |
| 24 | A/duck/Vietnam/NCVD-391/2009 | 2.3.4 | 8 | 2 | 4 | 4 | 4 | 4 | 8 | 2 | 8 | 4 | 8 | 2 | 4 | 4 | 32 | 8 | 64 | 32 | 16 | 8 | | 4 | | 1 | | 8 | | 4 | | 2 | | 0.5 | | 2 | | 0.5 | | 16 | | 64 | | 128 | |  |  |  |
| 25 | A/Japanese-white-eye/Hong Kong/1038/06 | 2.3.4 | 2 | 0.5 | 2 | 0.5 | 2 | 2 | 4 | 1 | 8 | 1 | 4 | 1 | 1 | 4 | 32 | 8 | 64 | 64 | 32 | 2 | | 2 | | 2 | | 1 | | 1 | | 1 | | 0.25 | | 2 | | 0.13 | | 8 | | 64 | | 64 | |  |  |  |
| 26 | A/chicken/India/NIV33487/2006 (IBCDC-RG7) | 2.3.4 | 4 | 8 | 8 | 2 | 4 | 2 | 2 | 2 | 2 | 2 | 2 | 16 | 8 | 8 | 32 | 16 | 64 | 64 | 64 | 8 | | 4 | | 2 | | 8 | | 1 | | 4 | | 0.25 | | 8 | | 1 | | 16 | | 64 | | 128 | |  |  |  |
| 27 | A/Anhui/1/2005 | 2.3.4 | 2 | 1 | 1 | 4 | 1 | 4 | 4 | 8 | 1 | 2 | 4 | 2 | 4 | 16 | 128 | 16 | 128 | 128 | 64 | 4 | | 2 | | 2 | | 1 | | 4 | | 1 | | 0.13 | | 8 | | 0.13 | | 16 | | 64 | | 128 | |  |  |  |
| 28 | A/duck/Vietnam/NCVD-293/2009 | 2.3.4.1 | 32 | 8 | 8 | 8 | 8 | 16 | 16 | 8 | 16 | 4 | 8 | 32 | 8 | 16 | 128 | 32 | 256 | 128 | 64 | 2 | | 4 | | 4 | | 4 | | 4 | | 4 | | 1 | | 8 | | 2 | | 16 | | 64 | | 128 | |  |  |  |
| 29 | A/chicken/Vietnam/NCVD-35/2008 | 2.3.4.2 | 8 | 4 | 8 | 0.5 | 8 | 32 | 16 | 4 | 16 | 4 | 8 | 2 | 8 | 4 | 32 | 4 | 32 | 64 | 64 | 4 | | 4 | | 2 | | 1 | | 4 | | 4 | | 0.25 | | 1 | | 1 | | 16 | | 64 | | 128 | |  |  |  |
| 30 | A/chicken/Vietnam/NCVD-279/2009 | 2.3.4.3 | 16 | 8 | 8 | 4 | 16 | 32 | 16 | 4 | 2 | 2 | 4 | 4 | 8 | 8 | 64 | 16 | 64 | 64 | 32 | 4 | | 8 | | 2 | | 4 | | 4 | | 4 | | 0.25 | | 4 | | 1 | | 16 | | 64 | | 128 | |  |  |  |
| 31 | A/chicken/Vietnam/NCVD-016/2008 | 7.1 | 64 | 8 | 8 | 32 | 16 | 64 | 32 | 8 | 32 | 8 | 8 | 32 | 16 | 8 | 128 | 32 | 256 | 128 | 64 | 16 | | 16 | | 16 | | 64 | | 16 | | 8 | | 16 | | 32 | | 16 | | 1 | | 8 | | 8 | |  |  |  |
| 32 | A/chicken/Vietnam/NCVD-016/2008 (IDCDC-RG12) | 7.1 | 64 | 8 | 4 | 16 | 16 | 16 | 16 | 2 | 16 | 4 | 8 | 16 | 8 | 4 | 64 | 16 | 256 | 128 | 32 | 32 | | 8 | | 8 | | 32 | | 8 | | 8 | | 4 | | 4 | | 4 | | 1 | | 1 | | 8 | |  |  |  |
| 33 | A/chicken/Vietnam/NCVD-3/2008 (IDCDC-RG25A) | 7.1 | 64 | 8 | 8 | 8 | 16 | 16 | 8 | 4 | 16 | 2 | 8 | 16 | 4 | 2 | 32 | 16 | 64 | 64 | 32 | 16 | | 4 | | 8 | | 32 | | 8 | | 16 | | 8 | | 8 | | 4 | | 1 | | 4 | | 1 | |  |  |  |
| 34 | A/Goose/Vietnam/113/2001 | 3 | 8 | 4 | 2 | 8 | 2 | 1 | 4 | 4 | 8 | 4 | 8 | 32 | 8 | 8 | 128 | 32 | 128 | 64 | 32 | 32 | | 4 | | 8 | | 64 | | 2 | | 8 | | 4 | | 2 | | 1 | | 16 | | 32 | | 128 | |  |  |  |
|  | **TEST ANTIGENS** |  |  |  |  |  |  |  |  |  |  |  |  |  |  |  |  |  |  |  |  |  | |  | |  | |  | |  | |  | |  | |  | |  | |  | |  | |  | |  |  |  |
| 35 | A/duck/Vietnam/NCVD-1/2002 | 5 | 1 | 4 | 2 | 1 | 4 | 1 | 4 | 1 | 1 | 1 | 2 | 0.5 | 4 | 1 | 16 | 8 | 32 | 64 | 16 | 4 | | 2 | | 2 | | 8 | | 2 | | 2 | | 0.13 | | 16 | | 0.25 | | 4 | | 32 | | 64 | |  |  |  |
| 36 | A/Whooper Swan/Mongolia/244/2005 | 2.2 | 4 | 4 | 1 | 16 | 2 | 2 | 2 | 2 | 1 | 2 | 2 | 16 | 4 | 2 | 64 | 16 | 64 | 16 | 4 | 8 | | 8 | | 4 | | 32 | | 2 | | 4 | | 1 | | 32 | | 2 | | 8 | | 64 | | 128 | |  |  |  |
| 37 | A/duck/Vietnam/10/2005 | 2.3.2 | 2 | 0.5 | 1 | 0.5 | 0.5 | 1 | 4 | 1 | 2 | 2 | 1 | 0.5 | 4 | 0.5 | 2 | 2 | 32 | 32 | 16 | 4 | | 2 | | 1 | | 2 | | 1 | | 2 | | 0.5 | | 4 | | 0.5 | | 4 | | 16 | | 32 | |  |  |  |
| 38 | A/duck/Vietnam/NCVD-471/2010 | 2.3.4.1 | 8 | 0.5 | 4 | 4 | 4 | 4 | 16 | 4 | 2 | 4 | 8 | 0.5 | 4 | 4 | 32 | 8 | 64 | 32 | 16 | 4 | | 4 | | 0.5 | | 4 | | 8 | | 2 | | 0.25 | | 1 | | 0.25 | | 4 | | 16 | | 64 | |  |  |  |
| 39 | A/duck/Vietnam/NCVD-472/2010 | 2.3.4.2 | 8 | 0.5 | 4 | 2 | 4 | 4 | 16 | 4 | 16 | 16 | 8 | 8 | 4 | 4 | 32 | 16 | 256 | 32 | 16 | 8 | | 8 | | 2 | | 4 | | 8 | | 2 | | 1 | | 1 | | 0.5 | | 16 | | 64 | | 128 | |  |  |  |
| 40 | A/duck/Vietnam/NCVD-19/2003 | 1 | 2 | 4 | 4 | 1 | 8 | 2 | 16 | 4 | 4 | 4 | 8 | 1 | 8 | 8 | 64 | 16 | 64 | 64 | 32 | 4 | | 8 | | 2 | | 32 | | 8 | | 8 | | 0.5 | | 32 | | 2 | | 4 | | 64 | | 64 | |  |  |  |
| 41 | A/chicken/Vietnam/NCVD-426/2009 | 1.1 | 4 | 0.5 | 2 | 2 | 16 | 8 | 16 | 4 | 8 | 16 | 8 | 2 | 2 | 4 | 64 | 32 | 128 | 64 | 16 | 4 | | 4 | | 8 | | 32 | | 8 | | 4 | | 1 | | 16 | | 4 | | 16 | | 64 | | 128 | |  |  |  |
| 42 | A/chicken/Vietnam/NCVD-427/2009 | 1.1 | 4 | 0.5 | 2 | 4 | 16 | 16 | 16 | 4 | 16 | 16 | 8 | 4 | 1 | 16 | 64 | 16 | 256 | 64 | 32 | 8 | | 8 | | 8 | | 16 | | 4 | | 4 | | 2 | | 8 | | 8 | | 16 | | 64 | | 128 | |  |  |  |
| 43 | A/duck/Vietnam/NCVD-430/2010 | 1.1 | 4 | 0.25 | 1 | 8 | 16 | 16 | 16 | 2 | 16 | 16 | 4 | 4 | 2 | 8 | 32 | 16 | 256 | 64 | 32 | 8 | | 8 | | 4 | | 32 | | 4 | | 4 | | 2 | | 16 | | 8 | | 16 | | 64 | | 128 | |  |  |  |
| 44 | A/duck/Vietnam/NCVD-1276/2012 | 1.1 | 8 | 0.5 | 4 | 4 | 16 | 32 | 16 | 4 | 16 | 8 | 8 | 4 | 2 | 4 | 64 | 16 | 128 | 64 | 16 | 8 | | 4 | | 8 | | 16 | | 4 | | 4 | | 1 | | 8 | | 4 | | 16 | | 64 | | 128 | |  |  |  |
| 45 | A/duck/Vietnam/NCVD-1279/2012 | 1.1 | 2 | 0.5 | 4 | 4 | 16 | 16 | 32 | 2 | 16 | 8 | 8 | 2 | 1 | 8 | 32 | 32 | 256 | 64 | 32 | 4 | | 4 | | 4 | | 32 | | 8 | | 8 | | 2 | | 16 | | 8 | | 16 | | 64 | | 128 | |  |  |  |
| 46 | A/chicken/Vietnam/NCVD-1277/2012 | 1.1 | 2 | 0.5 | 2 | 4 | 8 | 16 | 32 | 4 | 16 | 8 | 4 | 2 | 1 | 8 | 64 | 32 | 256 | 128 | 16 | 4 | | 4 | | 8 | | 32 | | 8 | | 8 | | 4 | | 8 | | 4 | | 16 | | 64 | | 128 | |  |  |  |
| 47 | A/chicken/Vietnam/NCVD-876/2011 | 1.1.1 | 2 | 0.25 | 1 | 2 | 8 | 8 | 16 | 4 | 8 | 8 | 4 | 2 | 1 | 8 | 64 | 16 | 128 | 64 | 32 | 4 | | 8 | | 8 | | 16 | | 8 | | 4 | | 2 | | 8 | | 8 | | 16 | | 64 | | 128 | |  |  |  |
| 48 | A/duck/Vietnam/NCVD-827/2011 | 1.1.1 | 2 | 0.25 | 1 | 2 | 16 | 16 | 16 | 4 | 8 | 4 | 8 | 4 | 1 | 4 | 64 | 16 | 128 | 64 | 32 | 8 | | 8 | | 8 | | 32 | | 8 | | 4 | | 2 | | 16 | | 8 | | 16 | | 64 | | 128 | |  |  |  |
| 49 | A/duck/Vietnam/NCVD-0193/2012 | 1.1.2 | 8 | 1 | 2 | 2 | 16 | 16 | 32 | 4 | 16 | 8 | 8 | 2 | 2 | 8 | 64 | 16 | 256 | 64 | 32 | 4 | | 4 | | 8 | | 32 | | 8 | | 2 | | 0.5 | | 4 | | 4 | | 16 | | 64 | | 128 | |  |  |  |
| 50 | A/duck/Vietnam/NCVD-2153/2012 | 1.1.2 | 4 | 1 | 2 | 1 | 32 | 8 | 32 | 4 | 16 | 8 | 8 | 2 | 1 | 8 | 64 | 16 | 128 | 64 | 32 | 4 | | 8 | | 8 | | 16 | | 4 | | 8 | | 2 | | 8 | | 8 | | 16 | | 64 | | 128 | |  |  |  |
| 51 | A/duck/Vietnam/NCVD-0139/2013 | 1.1.2 | 8 | 2 | 2 | 1 | 32 | 16 | 32 | 2 | 8 | 4 | 8 | 2 | 2 | 4 | 32 | 16 | 256 | 64 | 32 | 4 | | 8 | | 4 | | 16 | | 8 | | 8 | | 1 | | 8 | | 8 | | 16 | | 64 | | 128 | |  |  |  |
| 52 | A/duck/Vietnam/NCVD-2722/2013 | 1.1.2 | 16 | 4 | 4 | 2 | 16 | 32 | 32 | 4 | 16 | 8 | 8 | 4 | 2 | 8 | 64 | 16 | 256 | 64 | 32 | 8 | | 4 | | 8 | | 32 | | 8 | | 4 | | 2 | | 16 | | 4 | | 16 | | 64 | | 128 | |  |  |  |
| 53 | A/duck/Vietnam/NCVD-2721/2013 | 1.1.2 | 8 | 1 | 4 | 1 | 32 | 16 | 32 | 4 | 8 | 8 | 8 | 4 | 2 | 8 | 64 | 16 | 128 | 64 | 32 | 8 | | 4 | | 8 | | 32 | | 8 | | 4 | | 2 | | 16 | | 4 | | 16 | | 64 | | 128 | |  |  |  |
| 54 | A/chicken/Vietnam/NCVD-2750/2013 | 1.1.2 | 8 | 1 | 2 | 2 | 16 | 8 | 32 | 4 | 16 | 8 | 8 | 2 | 1 | 4 | 64 | 4 | 128 | 64 | 32 | 4 | | 8 | | 8 | | 32 | | 8 | | 8 | | 4 | | 16 | | 8 | | 16 | | 64 | | 128 | |  |  |  |
| 55 | A/chicken/Vietnam/NCVD-2716/2013 | 1.1.2 | 8 | 2 | 2 | 1 | 8 | 8 | 32 | 4 | 16 | 8 | 8 | 1 | 1 | 8 | 64 | 16 | 128 | 64 | 32 | 8 | | 4 | | 8 | | 32 | | 4 | | 4 | | 1 | | 8 | | 4 | | 16 | | 64 | | 128 | |  |  |  |
| 56 | A/chicken/Vietnam/NCVD-1096/2013 | 7.2 | 64 | 8 | 8 | 32 | 32 | 32 | 32 | 8 | 16 | 16 | 16 | 16 | 16 | 16 | 128 | 32 | 128 | 128 | 64 | 32 | | 16 | | 16 | | 64 | | 16 | | 32 | | 16 | | 32 | | 16 | | 16 | | 64 | | 128 | |  |  |  |
| 57 | A/chicken/Vietnam/NCVD-93/2008 | 7.2 | 64 | 8 | 8 | 32 | 16 | 16 | 32 | 8 | 16 | 16 | 16 | 32 | 16 | 16 | 128 | 32 | 256 | 128 | 64 | 64 | | 16 | | 16 | | 32 | | 8 | | 16 | | 8 | | 16 | | 16 | | 16 | | 64 | | 128 | |  |  |  |
| 58 | A/chicken/Vietnam/NCVD-1088/2013 | 7.2 | 64 | 8 | 8 | 32 | 32 | 32 | 32 | 8 | 16 | 16 | 8 | 32 | 16 | 16 | 64 | 32 | 128 | 128 | 64 | 64 | | 16 | | 16 | | 64 | | 16 | | 32 | | 16 | | 32 | | 16 | | 16 | | 64 | | 128 | |  |  |  |
| 59 | A/chicken/Vietnam/NCVD-1104/2013 | 7.2 | 64 | 8 | 4 | 16 | 32 | 32 | 32 | 8 | 32 | 16 | 16 | 32 | 16 | 16 | 128 | 32 | 256 | 128 | 64 | 64 | | 16 | | 16 | | 64 | | 16 | | 16 | | 8 | | 32 | | 16 | | 16 | | 64 | | 128 | |  |  |  |
| 60 | A/chicken/Vietnam/NCVD-449/2010 | 2.3.2.1 | 16 | 1 | 2 | 8 | 4 | 32 | 16 | 2 | 16 | 8 | 4 | 4 | 1 | 1 | 4 | 2 | 16 | 16 | 8 | 8 | | 1 | | 4 | | 32 | | 8 | | 8 | | 2 | | 2 | | 1 | | 16 | | 64 | | 128 | |  |  |  |
| 61 | A/chicken/Vietnam/NCVD-460/2010 | 2.3.2.1 | 32 | 2 | 2 | 8 | 2 | 32 | 16 | 4 | 32 | 8 | 8 | 4 | 2 | 1 | 8 | 1 | 16 | 8 | 8 | 16 | | 2 | | 4 | | 16 | | 8 | | 8 | | 2 | | 2 | | 2 | | 16 | | 64 | | 128 | |  |  |  |
| 62 | A/duck/Vietnam/NCVD-1171/2012 | 2.3.2.1a | 16 | 1 | 4 | 8 | 4 | 4 | 16 | 4 | 2 | 1 | 1 | 4 | 2 | 2 | 16 | 4 | 8 | 2 | 2 | 2 | | 8 | | 4 | | 32 | | 8 | | 4 | | 0.5 | | 16 | | 2 | | 8 | | 32 | | 64 | |  |  |  |
| 63 | A/duck/Vietnam/NCVD-1207/2012 | 2.3.2.1a | 16 | 1 | 4 | 2 | 1 | 32 | 4 | 8 | 8 | 1 | 1 | 2 | 0.5 | 4 | 16 | 2 | 4 | 2 | 2 | 2 | | 8 | | 4 | | 32 | | 4 | | 16 | | 0.5 | | 4 | | 2 | | 16 | | 64 | | 128 | |  |  |  |
| 64 | A/chicken/Vietnam/NCVD-1223/2012 | 2.3.2.1a | 16 | 1 | 2 | 4 | 2 | 32 | 8 | 2 | 4 | 2 | 1 | 2 | 1 | 16 | 8 | 2 | 16 | 4 | 4 | 4 | | 8 | | 4 | | 32 | | 8 | | 8 | | 1 | | 8 | | 1 | | 16 | | 64 | | 128 | |  |  |  |
| 65 | A/chicken/Vietnam/NCVD-1247/2012 | 2.3.2.1a | 8 | 0.5 | 2 | 4 | 2 | 64 | 4 | 2 | 4 | 2 | 1 | 2 | 1 | 16 | 16 | 2 | 8 | 4 | 2 | 4 | | 4 | | 8 | | 16 | | 8 | | 4 | | 1 | | 8 | | 2 | | 16 | | 64 | | 128 | |  |  |  |
| 66 | A/chicken/Vietnam/NCVD-1255/2012 | 2.3.2.1a | 8 | 1 | 4 | 8 | 2 | 32 | 4 | 4 | 8 | 1 | 1 | 1 | 1 | 16 | 32 | 4 | 8 | 8 | 4 | 4 | | 8 | | 8 | | 32 | | 8 | | 4 | | 0.5 | | 8 | | 4 | | 16 | | 64 | | 128 | |  |  |  |
| 67 | A/chicken/Vietnam/NCVD-1488/2012 | 2.3.2.1a | 16 | 2 | 4 | 4 | 4 | 32 | 4 | 4 | 8 | 2 | 2 | 1 | 0.5 | 16 | 8 | 2 | 16 | 8 | 4 | 16 | | 4 | | 8 | | 32 | | 8 | | 8 | | 1 | | 4 | | 1 | | 16 | | 64 | | 128 | |  |  |  |
| 68 | A/duck/Vietnam/NCVD-1494/2012 | 2.3.2.1a | 8 | 1 | 4 | 2 | 4 | 32 | 8 | 2 | 8 | 1 | 2 | 4 | 1 | 16 | 8 | 4 | 32 | 8 | 8 | 8 | | 8 | | 8 | | 32 | | 8 | | 8 | | 0.5 | | 4 | | 1 | | 16 | | 64 | | 128 | |  |  |  |
| 69 | A/chicken/Vietnam/NCVD-1963/2012 | 2.3.2.1a | 16 | 1 | 4 | 4 | 4 | 32 | 8 | 2 | 4 | 4 | 1 | 2 | 1 | 16 | 16 | 4 | 16 | 8 | 4 | 4 | | 8 | | 8 | | 32 | | 8 | | 4 | | 0.5 | | 8 | | 2 | | 16 | | 64 | | 128 | |  |  |  |
| 70 | A/chicken/Vietnam/NCVD-2749/2013 | 2.3.2.1a | 16 | 1 | 4 | 4 | 4 | 32 | 16 | 8 | 2 | 2 | 2 | 4 | 2 | 16 | 16 | 2 | 16 | 16 | 4 | 4 | | 8 | | 8 | | 32 | | 16 | | 8 | | 1 | | 8 | | 2 | | 16 | | 64 | | 128 | |  |  |  |
| 71 | A/duck/Vietnam/NCVD-1161/2011 | 2.3.2.1b | 32 | 8 | 8 | 32 | 1 | 32 | 8 | 2 | 8 | 0.5 | 2 | 4 | 1 | 2 | 8 | 1 | 16 | 2 | 2 | 4 | | 4 | | 4 | | 32 | | 8 | | 8 | | 0.5 | | 8 | | 8 | | 16 | | 64 | | 128 | |  |  |  |
| 72 | A/duck/Vietnam/NCVD129-7/2011 | 2.3.2.1b | 32 | 8 | 4 | 8 | 2 | 8 | 4 | 1 | 2 | 2 | 4 | 4 | 2 | 0.5 | 2 | 2 | 128 | 8 | 2 | 4 | | 8 | | 8 | | 64 | | 8 | | 8 | | 1 | | 32 | | 8 | | 4 | | 32 | | 64 | |  |  |  |
| 73 | A/duck/Vietnam/NCVD-1283/2012 | 2.3.2.1b | 64 | 8 | 8 | 32 | 8 | 32 | 8 | 2 | 16 | 4 | 4 | 4 | 1 | 2 | 2 | 1 | 32 | 32 | 16 | 8 | | 16 | | 16 | | 64 | | 16 | | 32 | | 8 | | 16 | | 16 | | 16 | | 64 | | 128 | |  |  |  |
| 74 | A/chicken/Vietnam/NCVD-1437/2012 | 2.3.2.1b | 64 | 8 | 8 | 32 | 32 | 32 | 8 | 2 | 16 | 4 | 4 | 8 | 2 | 2 | 4 | 1 | 32 | 16 | 8 | 16 | | 8 | | 16 | | 32 | | 8 | | 32 | | 8 | | 32 | | 8 | | 16 | | 64 | | 128 | |  |  |  |
| 75 | A/duck/Vietnam/NCVD-1463/2012 | 2.3.2.1b | 64 | 8 | 8 | 32 | 2 | 32 | 4 | 2 | 16 | 1 | 1 | 4 | 1 | 0.5 | 4 | 1 | 16 | 4 | 4 | 2 | | 16 | | 16 | | 32 | | 16 | | 4 | | 16 | | 32 | | 16 | | 16 | | 64 | | 128 | |  |  |  |
| 76 | A/duck/Vietnam/NCVD-1497/2012 | 2.3.2.1b | 32 | 8 | 8 | 32 | 2 | 32 | 8 | 2 | 16 | 2 | 1 | 2 | 0.5 | 2 | 4 | 2 | 16 | 8 | 4 | 4 | | 8 | | 8 | | 32 | | 8 | | 8 | | 16 | | 16 | | 16 | | 16 | | 64 | | 128 | |  |  |  |
| 77 | A/duck/Vietnam/NCVD-1940/2012 | 2.3.2.1b | 64 | 8 | 8 | 32 | 1 | 32 | 8 | 2 | 16 | 1 | 1 | 2 | 1 | 1 | 8 | 1 | 4 | 8 | 2 | 2 | | 8 | | 8 | | 64 | | 8 | | 32 | | 8 | | 32 | | 8 | | 16 | | 64 | | 128 | |  |  |  |
| 78 | A/chicken/Vietnam/NCVD-2693/2013 | 2.3.2.1b | 16 | 8 | 8 | 32 | 4 | 32 | 8 | 4 | 16 | 1 | 1 | 4 | 2 | 2 | 8 | 2 | 16 | 4 | 4 | 4 | | 16 | | 16 | | 64 | | 16 | | 32 | | 16 | | 16 | | 16 | | 16 | | 64 | | 128 | |  |  |  |
| 79 | A/duck/Vietnam/NCVD-1547/2012 | 2.3.2.1c | 16 | 4 | 8 | 16 | 1 | 32 | 8 | 4 | 2 | 1 | 0.5 | 2 | 4 | 2 | 4 | 4 | 2 | 1 | 1 | 1 | | 4 | | 4 | | 16 | | 4 | | 4 | | 2 | | 8 | | 4 | | 16 | | 64 | | 128 | |  |  |  |
| 80 | A/duck/Vietnam/NCVD-1584/2012 | 2.3.2.1C | 32 | 8 | 4 | 16 | 2 | 8 | 8 | 4 | 2 | 0.5 | 0.5 | 4 | 2 | 1 | 16 | 4 | 2 | 1 | 1 | 1 | | 8 | | 4 | | 32 | | 8 | | 8 | | 1 | | 8 | | 4 | | 8 | | 64 | | 128 | |  |  |  |
| 81 | A/duck/Vietnam/NCVD-1869/2012 | 2.3.2.1c | 64 | 4 | 8 | 32 | 4 | 32 | 4 | 4 | 8 | 0.5 | 2 | 2 | 0.5 | 2 | 16 | 4 | 8 | 2 | 2 | 4 | | 4 | | 4 | | 16 | | 4 | | 8 | | 1 | | 4 | | 4 | | 16 | | 64 | | 128 | |  |  |  |
| 82 | A/duck/Vietnam/NCVD-1901/2012 | 2.3.2.1c | 64 | 4 | 8 | 32 | 1 | 32 | 4 | 2 | 4 | 0.5 | 1 | 2 | 1 | 1 | 8 | 4 | 4 | 2 | 2 | 2 | | 4 | | 4 | | 16 | | 4 | | 8 | | 1 | | 8 | | 4 | | 16 | | 64 | | 128 | |  |  |  |
| 83 | A/duck/Vietnam/NCVD-1905/2012 | 2.3.2.1c | 64 | 4 | 8 | 32 | 1 | 32 | 4 | 2 | 2 | 1 | 1 | 4 | 1 | 1 | 8 | 2 | 4 | 1 | 1 | 2 | | 4 | | 4 | | 16 | | 4 | | 8 | | 2 | | 8 | | 4 | | 16 | | 64 | | 128 | |  |  |  |
| 84 | A/duck/Vietnam/NCVD-1928/2012 | 2.3.2.1c | 64 | 4 | 8 | 32 | 2 | 64 | 2 | 4 | 4 | 1 | 0.5 | 4 | 2 | 4 | 8 | 2 | 4 | 2 | 1 | 1 | | 4 | | 8 | | 16 | | 8 | | 8 | | 2 | | 8 | | 4 | | 16 | | 64 | | 128 | |  |  |  |
| 85 | A/duck/Vietnam/NCVD-1933/2012 | 2.3.2.1c | 64 | 4 | 4 | 32 | 1 | 64 | 8 | 2 | 4 | 1 | 1 | 2 | 2 | 1 | 8 | 2 | 4 | 2 | 0.5 | 2 | | 4 | | 4 | | 16 | | 4 | | 8 | | 1 | | 8 | | 8 | | 16 | | 64 | | 128 | |  |  |  |
| 86 | A/duck/Vietnam/NCVD-1936/2012 | 2.3.2.1c | 64 | 2 | 4 | 32 | 1 | 64 | 8 | 4 | 4 | 1 | 1 | 2 | 1 | 2 | 8 | 4 | 4 | 2 | 2 | 2 | | 2 | | 8 | | 8 | | 4 | | 8 | | 1 | | 8 | | 4 | | 16 | | 64 | | 128 | |  |  |  |
| 87 | A/chicken/Vietnam/NCVD-1944/2012 | 2.3.2.1c | 64 | 4 | 8 | 32 | 2 | 64 | 4 | 4 | 4 | 1 | 1 | 2 | 0.5 | 2 | 16 | 2 | 4 | 2 | 1 | 2 | | 4 | | 8 | | 16 | | 4 | | 8 | | 1 | | 8 | | 4 | | 16 | | 64 | | 128 | |  |  |  |
| 88 | A/duck/Vietnam/NCVD-1968/2012 | 2.3.2.1c | 64 | 4 | 8 | 32 | 2 | 32 | 4 | 4 | 4 | 1 | 1 | 4 | 4 | 2 | 16 | 2 | 4 | 2 | 2 | 4 | | 2 | | 4 | | 32 | | 8 | | 8 | | 2 | | 16 | | 8 | | 16 | | 64 | | 128 | |  |  |  |
| 89 | A/duck/Vietnam/NCVD-2047/2012 | 2.3.2.1c | 64 | 4 | 8 | 32 | 2 | 32 | 4 | 2 | 4 | 1 | 0.5 | 2 | 2 | 2 | 16 | 4 | 8 | 4 | 2 | 4 | | 4 | | 4 | | 16 | | 4 | | 4 | | 1 | | 8 | | 4 | | 16 | | 64 | | 128 | |  |  |  |
| 90 | A/duck/Vietnam/NCVD-01-145/2013 | 2.3.2.1c | 64 | 4 | 8 | 16 | 1 | 64 | 4 | 2 | 8 | 1 | 0.5 | 4 | 1 | 1 | 8 | 2 | 2 | 2 | 1 | 2 | | 4 | | 4 | | 16 | | 4 | | 8 | | 2 | | 4 | | 4 | | 16 | | 64 | | 128 | |  |  |  |
| 91 | A/duck/Vietnam/NCVD-0170/2013 | 2.3.2.1c | 64 | 8 | 8 | 16 | 4 | 32 | 2 | 2 | 8 | 1 | 1 | 1 | 1 | 2 | 16 | 4 | 2 | 1 | 1 | 1 | | 4 | | 4 | | 16 | | 4 | | 8 | | 1 | | 8 | | 4 | | 16 | | 64 | | 128 | |  |  |  |
| 92 | A/Swiftlet/Vietnam/NCVD-3000/2013 | 2.3.2.1c | 64 | 8 | 8 | 32 | 2 | 32 | 4 | 2 | 4 | 2 | 2 | 1 | 1 | 2 | 16 | 4 | 8 | 2 | 2 | 4 | | 8 | | 4 | | 8 | | 4 | | 8 | | 1 | | 8 | | 4 | | 16 | | 64 | | 128 | |  |  |  |
| 93 | A/duck/Vietnam/NCVD-2709/2013 | 2.3.2.1c | 32 | 8 | 8 | 32 | 4 | 64 | 4 | 1 | 4 | 1 | 1 | 2 | 1 | 2 | 16 | 4 | 16 | 4 | 4 | 4 | | 4 | | 2 | | 8 | | 2 | | 4 | | 1 | | 8 | | 4 | | 16 | | 64 | | 128 | |  |  |  |
| 94 | A/duck/Vietnam/NCVD-2726/2013 | 2.3.2.1c | 32 | 8 | 8 | 32 | 2 | 64 | 8 | 1 | 4 | 1 | 0.5 | 2 | 2 | 2 | 16 | 4 | 4 | 4 | 4 | 2 | | 4 | | 2 | | 16 | | 4 | | 4 | | 1 | | 8 | | 4 | | 16 | | 64 | | 128 | |  |  |  |
| 95 | A/duck/Vietnam/NCVD-2727/2013 | 2.3.2.1c | 32 | 4 | 8 | 32 | 2 | 64 | 4 | 2 | 8 | 0.5 | 2 | 2 | 2 | 2 | 16 | 4 | 8 | 4 | 2 | 2 | | 4 | | 4 | | 16 | | 4 | | 4 | | 1 | | 16 | | 4 | | 16 | | 64 | | 128 | |  |  |  |
| 96 | A/duck/Vietnam/NCVD-2745/2013 | 2.3.2.1c | 32 | 4 | 8 | 16 | 2 | 64 | 4 | 1 | 4 | 0.5 | 1 | 4 | 2 | 2 | 8 | 2 | 4 | 4 | 2 | 4 | | 4 | | 2 | | 16 | | 2 | | 4 | | 2 | | 16 | | 4 | | 16 | | 64 | | 128 | |  |  |  |
| 97 | A/duck/Vietnam/NCVD-0177/2013 | 2.3.2.1c | 16 | 8 | 8 | 32 | 1 | 64 | 4 | 1 | 4 | 1 | 1 | 2 | 1 | 2 | 16 | 2 | 4 | 2 | 2 | 4 | | 8 | | 4 | | 16 | | 4 | | 4 | | 1 | | 8 | | 4 | | 16 | | 64 | | 128 | |  |  |  |
| 98 | A/duck/Vietnam/NCVD-2848/2013 | 2.3.2.1c | 32 | 4 | 8 | 32 | 1 | 32 | 4 | 1 | 4 | 1 | 0.5 | 1 | 0.5 | 2 | 16 | 4 | 4 | 2 | 1 | 2 | | 4 | | 4 | | 16 | | 2 | | 8 | | 1 | | 8 | | 4 | | 16 | | 64 | | 128 | |  |  |  |
| 99 | A/Swiftlet/Vietnam/NCVD-3000/2013 | 2.3.2.1c | 64 | 8 | 8 | 32 | 2 | 32 | 4 | 2 | 8 | 1 | 1 | 4 | 2 | 1 | 16 | 2 | 16 | 2 | 2 | 4 | | 2 | | 4 | | 16 | | 4 | | 8 | | 2 | | 8 | | 4 | | 16 | | 64 | | 128 | |  |  |  |
| 100 | A/chicken/Vietnam/NCVD-14-A318/2014 | 2.3.2.1c | 32 | 4 | 8 | 32 | 4 | 32 | 4 | 2 | 8 | 0.5 | 1 | 1 | 2 | 2 | 16 | 2 | 2 | 1 | 0.5 | 1 | | 4 | | 4 | | 16 | | 4 | | 4 | | 1 | | 8 | | 4 | | 16 | | 64 | | 128 | |  |  |  |
| 101 | A/duck/Vietnam/NCVD-14-A332/2014 | 2.3.2.1c | 8 | 2 | 8 | 32 | 32 | 32 | 8 | 2 | 8 | 2 | 4 | 8 | 2 | 1 | 16 | 2 | 4 | 4 | 1 | 2 | | 4 | | 4 | | 16 | | 4 | | 4 | | 4 | | 8 | | 4 | | 16 | | 64 | | 128 | |  |  |  |

**Supplemental Table 2**: HI fold difference using ferret antisera

| **REFERENCE ANTIGENS** | | **Clade** | **GD/1** | **VN/ 1203** | **CB/ R0405050** | **IN5/2005** | **TK1/2005** | **EG/ 321** | **N03072/2010 RG29** | **WS/MG/244** | **HB/1/2010 RG30** | **BS/HK/1161/10** | **NCVD-672/ 2011** | **HK/ 6841/ 2010** | **NCVD-1544/ 2012** | **NCVD-1584/2012** | **AH/1/2005** | **NCVD-293/ 2009** | **NCVD-3/2008** |
| --- | --- | --- | --- | --- | --- | --- | --- | --- | --- | --- | --- | --- | --- | --- | --- | --- | --- | --- | --- |
| 1 | A/goose/Guangdong/1/1996 | 0 | 1 | 1 | 2 | 32 | 8 | 8 | 8 | 1 | 4 | 4 | 2 | 8 | 1 | 2 | 2 | 4 | 32 |
| 2 | A/Vietnam/1203/2004 | 1 | 8 | 1 | 2 | 64 | 32 | 32 | 64 | 4 | 4 | 16 | 32 | 64 | 4 | 16 | 8 | 16 | 128 |
| 3 | A/Cambodia/R0405050/2007 | 1.1 | 16 | 8 | 1 | 128 | 64 | 64 | 128 | 8 | 16 | 32 | 32 | 16 | 8 | 16 | 64 | 16 | 128 |
| 4 | A/Indonesia/5/2005 | 2.1.3.2 | 64 | 4 | 32 | 1 | 16 | 1 | 64 | 16 | 32 | 4 | 4 | 4 | 1 | 2 | 2 | 32 | 128 |
| 5 | A/turkey/Turkey/1/2005 | 2.2.1 | 4 | 4 | 4 | 4 | 1 | 1 | 4 | 1 | 2 | 4 | 2 | 2 | 0.25 | 0.5 | 4 | 16 | 128 |
| 6 | A/Egypt/321-Namru3/2007 | 2.2.1 | 4 | 4 | 64 | 8 | 4 | 1 | 8 | 1 | 8 | 16 | 8 | 4 | 2 | 2 | 4 | 16 | 128 |
| 7 | A/Egypt/N03072/2010 (IDCDC-RG29) | 2.2.1 | 1 | 2 | 1 | 8 | 2 | 2 | 1 | 1 | 1 | 0.5 | 2 | 1 | 0.25 | 0.5 | 4 | 4 | 128 |
| 8 | A/Hubei/1/2010 (IDCDC-RG30) | 2.3.2.1a | 16 | 8 | 8 | 16 | 2 | 4 | 32 | 1 | 1 | 2 | 2 | 1 | 1 | 1 | 16 | 4 | 64 |
| 9 | A/barn-Swallow/Hong Kong/D10-1161/2010 | 2.3.2.1b | 64 | 64 | 32 | 32 | 4 | 8 | 64 | 2 | 2 | 1 | 1 | 2 | 2 | 8 | 32 | 8 | 128 |
| 10 | A/duck/Vietnam/NCVD-672/2011 | 2.3.2.1c | 64 | 32 | 8 | 32 | 8 | 4 | 128 | 4 | 1 | 4 | 1 | 8 | 2 | 2 | 8 | 32 | 64 |
| 11 | A/Hong Kong/6841/2010 | 2.3.2.1c | 64 | 32 | 32 | 16 | 2 | 8 | 32 | 2 | 1 | 2 | 2 | 1 | 1 | 2 | 16 | 32 | 128 |
| 12 | A/duck/Vietnam/NCVD-1544/2012 | 2.3.2.1c | 64 | 16 | 32 | 16 | 2 | 8 | 32 | 2 | 2 | 4 | 8 | 0.5 | 1 | 2 | 32 | 32 | 64 |
| 13 | A/duck/Vietnam/NCVD-1584/2012 | 2.3.2.1c | 64 | 32 | 32 | 16 | 2 | 8 | 64 | 2 | 2 | 4 | 4 | 1 | 1 | 1 | 16 | 32 | 128 |
| 14 | A/Anhui/1/2005 | 2.3.4 | 8 | 4 | 2 | 4 | 16 | 8 | 8 | 2 | 64 | 32 | 16 | 8 | 8 | 16 | 1 | 1 | 64 |
| 15 | A/duck/Vietnam/NCVD-293/2009 | 2.3.4.1 | 2 | 32 | 1 | 4 | 4 | 64 | 8 | 2 | 4 | 1 | 8 | 1 | 0.5 | 1 | 4 | 1 | 64 |
| 16 | A/chicken/Vietnam/NCVD-3/2008 (IDCDC-RG25A) | 7.1 | 16 | 32 | 16 | 256 | 32 | 64 | 64 | 16 | 64 | 4 | 8 | 32 | 4 | 8 | 128 | 16 | 1 |
| 17 | A/Whooper Swan/Mongolia/244/2005 (Ag8) | 2.2 | 4 | 8 | 4 | 16 | 2 | 8 | 4 | 1 | 1 | 4 | 4 | 2 | 0.25 | 4 | 4 | 32 | 64 |
|  | **TEST ANTIGENS** |  |  |  |  |  |  |  |  |  |  |  |  |  |  |  |  |  |  |
| 18 | A/duck/Vietnam/NCVD-016/2007 | 1.1 | 8 | 8 | 2 | 64 | 32 | 64 | 128 | 8 | 4 | 16 | 32 | 8 | 4 | 16 | 64 | 16 | 128 |
| 19 | A/chicken/Vietnam/NCVD-1192/2012 | 1.1 | 64 | 4 | 2 | 64 | 32 | 64 | 128 | 16 | 64 | 16 | 32 | 8 | 8 | 16 | 16 | 16 | 128 |
| 20 | A/duck/Vietnam/NCVD-19/2003 | 1 | 8 | 16 | 2 | 16 | 64 | 4 | 16 | 4 | 2 | 8 | 32 | 4 | 2 | 4 | 2 | 8 | 64 |
| 21 | A/Indonesia/5/2005 (CDC-RG2) | 2.1.3.2 | 64 | 4 | 4 | 1 | 4 | 0.5 | 64 | 8 | 32 | 4 | 32 | 2 | 1 | 4 | 2 | 16 | 32 |
| 22 | A/Egypt/321-Namru3/2007 (IDCDC-RG11) | 2.2.1 | 4 | 2 | 4 | 16 | 2 | 1 | 8 | 0.5 | 4 | 4 | 4 | 4 | 1 | 1 | 4 | 16 | 128 |
| 23 | A/duck/Vietnam/10/2005 | 2.3.2 | 4 | 4 | 4 | 16 | 8 | 8 | 32 | 2 | 2 | 4 | 2 | 4 | 0.25 | 2 | 1 | 8 | 32 |
| 24 | A/Common magpie/Hongkong/5052/2007 | 2.3.2.1 | 32 | 16 | 32 | 4 | 4 | 8 | 64 | 2 | 1 | 2 | 4 | 1 | 0.5 | 1 | 8 | 32 | 128 |
| 25 | A/chicken/Vietnam/NCVD-675/2011 | 2.3.2.1a | 32 | 2 | 8 | 64 | 2 | 4 | 64 | 1 | 1 | 1 | 1 | 4 | 0.25 | 1 | 8 | 32 | 32 |
| 26 | A/duck/Vietnam/NCVD-1207/2012 | 2.3.2.1a | 0 | 32 | 32 | 16 | 8 | 8 | 64 | 4 | 5.33 | 2 | 8 | 2 | 0.5 | 0.5 | 64 | 32 | 128 |
| 27 | A/duck/Vietnam/NCVD-1171/2012 | 2.3.2.1a | 4 | 32 | 32 | 32 | 4 | 8 | 64 | 4 | 2 | 4 | 2 | 2 | 1 | 4 | 64 | 32 | 128 |
| 28 | A/duck/Vietnam/NCVD-1163/2012 | 2.3.2.1b | 32 | 16 | 32 | 8 | 4 | 4 | 256 | 4 | 1 | 0.5 | 2 | 2 | 0.5 | 0.5 | 16 | 32 | 128 |
| 29 | A/duck/Vietnam/NCVD129-7/2011 | 2.3.2.1b | 32 | 64 | 16 | 32 | 4 | 8 | 64 | 2 | 8 | 2 | 2 | 2 | 2 | 1 | 16 | 32 | 64 |
| 30 | A/Barheaded goose/Mongolia/X53/2009 | 2.3.2.1 c | 64 | 32 | 32 | 8 | 2 | 8 | 32 | 1 | 1 | 2 | 2 | 1 | 0.5 | 1 | 32 | 32 | 128 |
| 31 | A/duck/Vietnam/NCVD-1648/2012 | 2.3.2.1C | 64 | 16 | 16 | 16 | 1 | 8 | 32 | 1 | 2 | 4 | 2 | 1 | 0.5 | 1 | 16 | 32 | 64 |
| 32 | A/duck/Vietnam/NCVD-471/2010 | 2.3.4 | 8 | 16 | 8 | 128 | 8 | 128 | 32 | 4 | 8 | 8 | 4 | 4 | 1 | 2 | 0.5 | 8 | 16 |
| 33 | A/duck/Vietnam/NCVD-003/2008 | 2.3.4 | 16 | 4 | 4 | 16 | 16 | 32 | 32 | 4 | 128 | 16 | 4 | 8 | 4 | 8 | 2 | 4 | 64 |
| 34 | A/duck/Vietnam/NCVD-391/2009 | 2.3.4 | 4 | 2 | 1 | 4 | 4 | 32 | 16 | 2 | 4 | 4 | 4 | 2 | 1 | 2 | 2 | 1 | 64 |
| 35 | A/Japanese-white-eye/Hongkong/1038/06 | 2.3.4 | 2 | 2 | 2 | 4 | 8 | 32 | 16 | 2 | 8 | 16 | 2 | 2 | 4 | 2 | 4 | 1 | 64 |
| 36 | A/chicken/India/NIV33487/2006 (IBCDC-RG7) | 2.3.4 | 2 | 4 | 4 | 4 | 1 | 32 | 2 | 0.5 | 2 | 2 | 2 | 2 | 0.5 | 1 | 4 | 16 | 64 |
| 37 | A/chicken/Vietnam/NCVD-35/2008 | 2.3.4.2 | 8 | 16 | 4 | 16 | 64 | 128 | 128 | 8 | 128 | 16 | 8 | 4 | 2 | 4 | 8 | 2 | 32 |
| 38 | A/chicken/Vietnam/NCVD-279/2009 | 2.3.4.3 | 8 | 16 | 2 | 16 | 64 | 64 | 32 | 4 | 128 | 8 | 2 | 4 | 1 | 2 | 8 | 2 | 64 |
| 39 | A/goose/Vietnam/113/2001 | 3 | 2 | 16 | 2 | 64 | 128 | 64 | 16 | 4 | 4 | 16 | 2 | 8 | 4 | 4 | 128 | 32 | 32 |
| 40 | A/duck/Vietnam/NCVD-1/2002 | 5 | 2 | 1 | 4 | 8 | 4 | 2 | 4 | 1 | 2 | 8 | 2 | 4 | 1 | 2 | 1 | 4 | 32 |
| 41 | A/chicken/Vietnam/NCVD-016/2008 | 7.1 | 32 | 32 | 16 | 256 | 64 | 64 | 128 | 16 | 128 | 8 | 8 | 32 | 8 | 8 | 128 | 64 | 16 |
| 42 | A/chicken/Vietnam/NCVD-016/2008(IDCDC-RG12) | 7.1 | 16 | 16 | 16 | 256 | 32 | 64 | 64 | 16 | 64 | 8 | 8 | 32 | 8 | 8 | 128 | 32 | 8 |
